# Little evidence for long‐term harm from antenatal corticosteroids in a population‐based very low birthweight young adult cohort

**DOI:** 10.1111/ppe.12886

**Published:** 2022-05-16

**Authors:** Brian A. Darlow, Sarah L. Harris, L. John Horwood

**Affiliations:** ^1^ Department of Paediatrics University of Otago Christchurch New Zealand; ^2^ Department of Psychological Medicine Christchurch Health and Development Study University of Otago Christchurch New Zealand

**Keywords:** antenatal corticosteroids, follow‐up, mental health, physical health, population‐based cohort study, preterm young adults, very low birthweight infants

## Abstract

**Background:**

Antenatal corticosteroids (ACS) given to mothers with anticipated very preterm delivery are widely used and improve infant outcomes. Follow‐up studies of the first trials of ACS have shown no adverse effects, but recently there have been concerns about possible longer‐term harms.

**Objectives:**

We aimed to assess the relationship of ACS therapy to a range of physical health and welfare measures in a cohort of very low birthweight (VLBW; <1500 g) young adults.

**Methods:**

Population‐based cohort follow‐up study. All VLBW infants born in New Zealand in 1986 were included in a prospective audit of retinopathy of prematurity. Perinatal data collection included information on ACS. At 26–30 years, 250 of 323 (77%) survivors participated, 58% having received ACS, with 229 assessed in one centre, including cardiovascular, metabolic, respiratory and neurocognitive measures. Differences in outcome between those receiving/not receiving ACS were summarised by the mean difference for continuous outcomes supplemented by Cohen’s d as a standardised measure of effect size (ES), and risk ratios (RRI) for dichotomous outcomes, adjusted for relevant covariates using generalised linear regression methods.

**Results:**

There were no or minimal adverse effects of receipt of ACS versus no receipt across a range of health and welfare outcomes, both for the full cohort (adjusted ES range *d* = 0.01–0.23; adjusted RR range 0.78–2.03) and for individuals with gestation <28 weeks (extremely preterm; EP), except for a small increase in rates of major depression. In EP adults, receipt of ACS was associated with a higher incidence of hypertension, but might have a small benefit for IQ.

**Conclusions:**

In this population‐based VLBW cohort, we detected minimal adverse outcomes associated with exposure to ACS by the third decade of life, a similar result to the 30‐year follow‐up of participants in the first ACS trial. However, further follow‐up is warranted.

**Synopsis**

**Study question**

Are there detectable adverse health or welfare outcomes after exposure to antenatal corticosteroids by the third decade of life?

**What’s already known**

Antenatal corticosteroids improve preterm infant survival and decrease morbidity. Many animal studies suggest harmful effects of antenatal corticosteroids in surviving offspring as do a few recent human studies of very preterm‐born adults. In contrast, a 30‐year follow‐up of individuals in the first randomised trial of antenatal corticosteroids found no adverse effects.

**What this study adds**

In a population‐based cohort follow‐up at mean age 28 years of adults born with very low birthweight, half of whom had received antenatal corticosteroids, we found no or minimal adverse effects of steroids across a range of health and welfare outcomes.

## 1 BACKGROUND

Antenatal corticosteroids (ACS) given to mothers for anticipated preterm delivery before 34 weeks are the standard of care in high‐income countries. ACS treatment is highly cost‐effective^1^ and reduces neonatal mortality, respiratory distress syndrome, intraventricular haemorrhage and possibly other morbidities.^2^, ^3^, ^4^ Despite these early benefits of ACS treatment for very preterm infants, concerns have been raised about potential long‐term adverse effects.^5^, ^6^, ^7^, ^8^

In their landmark 1972 Auckland Steroid Trial,^9^, ^10^ Liggins and Howie reported the outcomes for the first 282 women, from a final total of 1142, ^10^ randomised to intramuscular betamethasone or placebo, repeated after 48 h if delivery had not occurred. The hypothesis, based on the results of a foetal infusion of dexamethasone in a sheep model,^11^ was that ACS would reduce respiratory distress. This proved correct, leading to a change in clinical practice that has had a profound impact on preterm infant survival and outcomes.

In 1989, Barker reported that males with the lowest birthweights, and/or the lowest weight at 1 year of age, had the highest mortality in adulthood from ischaemic heart disease.^12^ Other reports followed, linking impaired foetal growth or low birthweight with increased risks of developing adult diseases including cardiovascular disease, diabetes mellitus and the metabolic syndrome.^13^ The concept that foetal and early life events shape (‘programme’) future development and health became known as the ‘developmental origins of health and disease’ (DOHaD).

More recently, concerns have been raised that the increased risk of non‐communicable disease seen with foetal malnutrition and the resultant increase in endogenous steroids might also occur with exogenous antenatal glucocorticoid exposure.^13^, ^14^ In animal models of ACS exposure, a range of neurological, cardiovascular and metabolic longer‐term adverse effects are seen.^15^, ^16^, ^17^, ^18^ However, these often follow higher doses of dexamethasone than used in humans and effects vary with species, sex and timing.^6^, ^13^, ^18^, ^19^

Human data examining the longer‐term effects of ACS exposure are limited. Children included in the Auckland Steroid Trial were followed at 4 and 6 years of age with no differences in cognitive or developmental outcomes between ACS exposed and non‐exposed.^20^, ^21^ At 30 years of age, 56% of survivors were assessed and no between‐group differences were found including for body size, adiposity, blood lipids, cortisol, blood pressure, diabetes, cardiovascular disease, bone mass, asthma, cognitive functioning, psychiatric morbidity and health‐related quality of life.^10^, ^22^, ^23^, ^24^ Follow‐up at 10 to 12 years of an Amsterdam RCT of ACS also found no between‐group differences in growth^25^ or cognitive or behavioural outcomes.^26^ Although reassuring, a major caveat with these findings is that surviving participants from the Auckland Steroid Trial had mean birthweight 2.3 kg, mean gestation 35 weeks and 31% had been born at term, hence potentially not representative of the many very preterm infants currently exposed to ACS.

We have assessed the relationship between ACS exposure (ACS+, ACS‐) and a range of health and welfare measures in a national cohort of 250 young adults born with very low birthweight (VLBW; <1500 g), compared with same age term‐born control subjects.

## 2 METHODS

### 2.1 Cohort selection

In 1986, all 413 VLBW infants born in New Zealand and admitted to a neonatal unit were included in a prospective audit of retinopathy of prematurity with 338 (82%) surviving to discharge home.

### 2.2 Exposure

Following birth, 170 perinatal items were collected, including whether or not the mother had received all or part of a course of ACS, the exposure of interest in this report.

### 2.3 Outcomes

The protocol for the most recent follow‐up of surviving members of the cohort at 26–30 years has been published^27^ as have the methods.^28^, ^29^ Briefly, 250 of 323 (77%) known VLBW survivors consented to take part in the study (questionnaire sample) with 229 (56% having received ACS) coming to one centre (Christchurch) for 2 days of investigations and assessments (Figure S1). Table S1 summarises the outcome measures used in this analysis, covering the domains of growth, metabolic, visual, cardiovascular and mental health and cognition (IQ). Respiratory outcomes in relation to ACS have been reported elsewhere.^30^, ^31^

### 2.4 Statistical analysis

Differences in perinatal/demographic factors between those receiving/not receiving ACS and between those assessed/not assessed were summarised by the mean or risk difference and 95% CI. Differences in outcome between those receiving or not receiving ACS were summarised by the mean difference (95% CI) for continuous outcomes and adjusted for a range of relevant covariates using linear regression methods. Mean differences were standardised using Cohen’s d to provide a common metric for effect size (ES) comparisons. By convention, a value of d = 0.20 is taken to imply a small ES and 0.50 as moderate.^32^ For dichotomous outcomes, ESs were summarised by the risk ratio (RR, 95% CI), with adjusted risk ratio (ARR) estimates calculated from logistic regressions using the method described by Norton. ^33^ The no‐steroid group was the reference for all comparisons. Where relevant, tests of sex by receipt of ACS interactions were conducted by testing the equality of ESs between sexes.

### 2.5 Missing data

Missing data ranged between 0% and 3% for most outcomes, with the exception of fat percentage, fat mass and fat‐free mass (4.8%), adult ADHD (8%) and reactive hyperaemic index (RHI) (13%). Comparisons of those assessed survivors with those not assessed (Table S2) showed weak evidence of selection bias, the assessed survivors having lower mean birthweight, a slight sex imbalance and the clinically assessed sample underrepresenting those with a history of neurosensory disability. To address issues of missing data and potential selection bias, the adjusted ES analyses also included an inverse probability weighting adjustment. Specifically, for each outcome a logistic regression model was first fitted to predict the probability of inclusion in the assessment sample from the measures in Table S2; the inverse of this probability was then used as the weight for each individual in the adjusted regression models.

### 2.6 Ethics approval

The study was approved by the Southern Health and Disability Ethics Committee, and all participants gave written informed consent.

## 3 RESULTS

Table 1 shows the demographics and perinatal characteristics of the VLBW cohort clinically assessed at 26–30 years by receipt of ACS, and Table S3 shows these data for the interview sample. Those receiving ACS had a lower gestation and higher birthweight z‐score, were less often small for gestational age and more often born in a level III (regional) centre. Fourteen participants were born at 34‐week gestation or more.

**TABLE 1:** Demographics and perinatal data of the VLBW cohort by receipt of antenatal corticosteroids (ACS)—clinically assessed sample

Measure	Receipt of antenatal corticosteroids (ACS)		Difference (95% CI)
	No (n = 100)	Yes (n = 129)	
Age at assessment (y), mean (SD)	28.4 (1.1)	28.4 (1.1)	−0.01 (−0.30, 0.27)
Male, %	47.0	42.6	−4.4 (−17.4, 8.6)
Māori/Pacific Island, %	36.0	27.1	−8.9 (−21.0, 3.3)
Gestation (weeks), mean (SD)	29.9 (2.9)	28.7 (2.0)	−1.16 (−1.81, −0.51)
<28‐week gestation, %	22.0	27.1	5.1 (−6.0, 16.3)
Birthweight (g), mean (SD)	1140 (228)	1127 (244)	−13.1 (−75.4, 49.1)
<1000 g, %	26.0	29.5	3.4 (−8.2, 15.1)
Birthweight z‐score, mean (SD)	−1.1 (1.4)	−0.5 (1.0)	0.59 (0.28, 0.90)
Small for gestational age^a^, %	42.0	23.3	−18.7 (−30.9, −6.6)
Respiratory distress syndrome, %	52.0	56.5	4.6 (−8.4, 17.6)
Bronchopulmonary dysplasia^b^, %	25.0	16.3	−8.7 (−19.3, 1.9)
Apgar score (5 min), median (IQR)	8.0 (6.5–9.0)	9.0 (7.0–9.0)	1.0 (0.41–1.59)
Retinopathy of prematurity, %	16.0	22.5	6.5 (−3.9, 16.7)
Necrotising enterocolitis, %	13.0	10.1	−2.9 (−11.3, 5.5)
Maternal preeclamptic toxaemia, %	27.0	22.5	−4.5 (−15.8, 6.8)
Born in a Level III centre, %	68.0	76.7	8.7 (−3.0, 20.4)
Maternal smoking during pregnancy, %	43.0	38.0	−5.0 (−17.8, 7.8)
Duration breast feeding (mths), mean (SD)	5.0 (5.7)	4.0 (5.8)	−0.91 (−2.46, 0.64)
Maternal age at childbirth, mean (SD)	25.9 (5.5)	26.0 (4.8)	0.05 (−1.31, 1.40)
Parental education^c^, mean (SD)	2.0 (0.8)	2.1 (0.8)	0.17 (−0.04, 0.39)

Abbreviations: IQR, interquartile range; SD, standard deviation.

^a^ Birthweight <10th centile.

^b^ Oxygen requirement at 36‐week post‐menstrual age.

^c^ Parental education scored in 3 levels (no formal qualifications/high school qualifications/tertiary qualifications) based on highest educational attainment of either parent.

Tables 2–6 summarise the analysis results for each outcome domain by receipt of ACS: adult growth outcomes (Table 2); blood pressure and metabolic data (Table 3); visual and cardiovascular outcomes (Table 4); mental health and substance use (Table 5); cognitive outcomes (IQ) and adult ADHD (Table 6). Tables S4–S8 show the same analyses by sex and Tables S9–S13 for those born EP.

**TABLE 2:** Adult growth outcomes by receipt antenatal corticosteroids (ACS)

Measure	Receipt of antenatal corticosteroids		Mean difference (95%CI)		Cohen’s d^a^
	No (n = 100)	Yes (n = 129)	Unadjusted	Adjusted^a^	
	Mean (SD)	Mean (SD)			
Height, cm	167.1 (8.4)	167.3 (9.3)	0.18 (−2.17, 2.54)	−0.22 (−1.86, 1.42)	0.02
Weight, kg	75.4 (18.2)	75.3 (20.2)	−0.07 (−5.20, 5.06)	−0.43 (−5.44, 4.58)	0.02
Body mass index, kg/m^2^	26.9 (6.0)	26.8 (6.4)	−0.08 (−1.74, 1.58)	−0.09 (−1.77, 1.60)	0.01
Waist circumference, cm	84.7 (14.6)	84.2 (15.1)	−0.43 (−4.34, 3.47)	−0.56 (−4.48, 3.35)	0.04
Hip circumference, cm	99.8 (11.8)	101.9 (12.5)	1.09 (−2.12, 4.30)	0.55 (−2.78, 3.87)	0.04
Waist/hip ratio	0.84 (0.09)	0.83 (0.08)	−0.01 (−0.04, 0.01)	−0.01 (−0.03, 0.01)	0.11
Body fat percentage, %	28.0 (10.8)	27.7 (10.6)	−0.34 (−3.24, 2.54)	−1.15 (−3.68, 1.38)	0.11
Fat mass, kg	22.4 (13.7)	22.3 (13.9)	−0.13 (−3.85, 3.59)	−0.78 (−4.51, 2.95)	0.06
Fat‐free mass, kg	53.0 (9.6)	53.2 (11.0)	0.18 (−2.63,2.99)	0.67 (−1.13, 2.46)	0.06
Total body water, kg	38.8 (7.0)	39.0 (8.1)	0.13 (−1.92, 2.19)	0.49 (−0.82, 1.80)	0.06

^a^ Adjusted for sex, ethnicity, birthweight z‐score, gestation, parental education, duration of breastfeeding and potential selection bias.

**TABLE 3:** Biomedical and metabolic indices by receipt of antenatal corticosteroids (ACS)

Measure	Receipt of ACS		Mean difference/RR (95% CI)		Cohen’s d^a^
	No	Yes	Unadjusted	Adjusted^a^	
Bloods, mean (sd)	(n = 99)	(n = 125)			
Fasting blood insulin (pmol/L)	76.2 (50.4)	71.4 (61.6)	−4.83 (−19.98, 10.32)	−2.61 (−18.74, 13.52)	0.04
Fasting blood glucose (mmol/L)	5.0 (0.5)	5.1 (0.5)	0.05 (−0.09, 0.18)	0.04 (−0.10, 0.18)	0.08
Haemoglobin A1c (mmol/mol)	31.6 (4.3)	32.2 (4.5)	0.65 (−0.53, 1.84)	1.03 (−0.17, 2.24)	0.23
HOMA‐IR	2.4 (1.7)	2.3 (2.0)	−0.11 (−0.61, 0.39)	−0.06 (−0.59, 0.48)	0.03

Blood pressure, mean (sd)	(n = 100)	(n = 129)			
Systolic BP (mm Hg)	114.4 (12.5)	113.4 (12.8)	−0.98 (−4.30, 2.35)	−0.81 (−4.10, 2.49)	0.06
Diastolic BP (mm Hg)	74.0 (9.8)	73.9 (9.0)	−0.13 (−2.60, 2.33)	0.11 (−2.34, 2.56)	0.01

Metabolic syndrome^b^, %	(n = 99)	(n = 126)	RR (95%CI)	ARR (95%CI)	
Elevated waist circumference	37.4	39.7	1.06 (0.76, 1.48)	1.05 (0.74, 1.49)	
Elevated triglycerides	24.2	17.6	0.73 (0.43, 1.21)	0.82 (0.46, 1.46)	
Reduced HDL‐C	37.4	39.7	1.13 (0.82, 1.57)	1.20 (0.86, 1.68)	
Elevated BP	19.2	16.7	0.87 (0.50, 1.52)	0.96 (0.52, 1.76)	
Elevated fasting glucose	11.1	18.4	1.65 (0.85, 3.23)	1.75 (0.85, 3.60)	
Metabolic syndrome	20.2	14.4	0.71 (0.40, 1.27)	0.78 (0.42, 1.44)	

Abbreviations: BP, blood pressure; HDL‐C, high‐density lipoprotein cholesterol; HOMA‐IR, homeostatic model assessment for insulin resistance.

^a^ Adjusted for sex, ethnicity, birthweight z‐score, gestation, duration of breast feeding, parental education and potential selection bias.

^b^ Symptoms of metabolic syndrome and elevated waist circumference defined according to International Diabetes Federation criteria.^57^

**TABLE 4:** Visual and cardiovascular outcomes by receipt of antenatal corticosteroids (ACS)

Measure	Receipt of ACS		Mean difference/RR (95% CI)		Cohen’s d^a^
	No	Yes	Unadjusted	Adjusted^a^	
Visual outcomes, %	(n = 100)	(n = 129)	RR (95%CI)	ARR (95%CI)	
Visual acuity (logMAR) >0.3 better eye	5.0	9.3	1.86 (0.68, 5.11)	1.95 (0.68, 5.60)	
Myopia >2.0 dioptres, better eye	15.2	11.9	0.79 (0.40, 1.53)	0.87 (0.41, 1.84)	
Hypermetropia >2.0 dioptres, better eye	0.0	3.2	‐	‐	
Astigmatism >2.0 dioptres, better eye	8.8	4.8	0.59 (0.21, 1.64)	0.79 (0.29, 2.15)	
Moderate visual impairment (any of the above)	22.0	23.3	1.06 (0.65, 1.72)	1.14 (0.67, 1.93)	

Heart structure and function, mean(sd)	(n = 99)	(n = 129)	Mean diff (95%CI)	Mean diff (95%CI)	
LV mass ‐ indexed BSA (g/m^2^)	90.4 (17.3)	89.1 (20.7)	−1.30 (−6.38, 3.78)	−0.36 (−5.58,4.87)	0.02
LVEDV ‐ indexed BSA (ml/m^2^)	59.1 (10.8)	57.8 (11.1)	−1.34 (−4.22, 1.54)	−1.01 (−4.08, 2.05)	0.09
LVESV ‐ indexed BSA (ml/m^2^)	21.2 (5.1)	20.5 (4.7)	−0.63 (−1.92, 0.65)	−0.48 (−1.82, 0.86)	0.10
LV elastance (mm Hg/ml)	3.31 (0.80)	3.42 (0.94)	0.11 (−0.13, 0.34)	0.14 (−0.10, 0.38)	0.16
Arterial elastance (mm Hg/ml)	1.82 (0.35)	1.86 (0.44)	0.04 (−0.07, 0.15)	0.06 (−0.05, 0.17)	0.15
Reactive hyperaemic index (RHI)^b^	1.90 (0.62)	1.91 (0.54)	0.01 (−0.16, 0.17)	−0.08 (−0.25, 0.09)	0.14
Cardiac output ‐ indexed BSA (L/min)	2.69 (0.63)	2.67 (0.64)	−0.02 (−0.19, 0.15)	0.01 (−0.17, 0.20)	0.02

Abbreviations: BSA, body surface area; logMAR, log of minimum angle of resolution; LV, left ventricular; LVEDV, LV end‐diastolic volume; LVESV, LV end‐systolic volume.

^a^ Adjusted for sex, ethnicity, birthweight z‐score, gestation, parental education, duration of breast feeding, history of ROP (visual outcomes only) and potential selection bias.

^b^ Sample sizes for RHI—no ACS (n = 89), ACS (n = 110).

**TABLE 5:** Adult mental health, substance use and antisocial behaviour outcomes by receipt of antenatal corticosteroids (ACS)

Measure	Receipt of ACS		Unadjusted RR (95% CI)	Adjusted RR (95% CI)^a^
	No (n = 109)	Yes (n = 141)		
Mental health (past 12 months), %				
Major depression	9.2	16.4	1.79 (0.89, 3.60)	2.03 (0.99, 4.18)
Suicidal ideation	7.3	6.4	0.87 (0.35, 2.18)	1.16 (0.41, 3.28)
Anxiety disorder	25.7	27.8	1.08 (0.71, 1.63)	1.08 (0.69,1.68)
Any of the above	32.1	37.6	1.17 (0.83, 1.65)	1.14 (0.78, 1.67)
Substance use/antisocial behaviour				
Daily smoker	33.0	27.7	0.84 (0.57, 1.22)	1.09 (0.71, 1.67)
Regular (weekly) binge drinking	18.4	13.5	0.73 (0.41, 1.31)	1.04 (0.55, 1.97)
Daily cannabis use	7.3	7.8	1.06 (0.44, 2.55)	1.06 (0.44, 2.56)
History of adult offending (>18 y)	28.4	17.7	0.62 (0.39, 0.99)	0.83 (0.50, 1.39)

^a^ Adjusted for sex, ethnicity, birthweight z‐score, gestation, parental education and potential selection bias.

**TABLE 6:** Adult IQ and attention deficit hyperactivity disorder (ADHD) by receipt of antenatal corticosteroids (ACS)

Measure	Receipt of ACS		Mean difference (95% CI)		Cohen’s d^a^
	No	Yes	Unadjusted	Adjusted^a^	
WASI II IQ scores, mean (SD)	(n = 100)	(n = 128)			
Verbal IQ	99.2 (11.7)	101.4 (15.3)	2.18 (−1.46, 5.82)	0.97 (−2.66, 4.59)	0.07
Perceptual IQ	98.4 (14.8)	100.6 (15.5)	2.13 (−1.89, 6.16)	2.62 (−1.93, 7.17)	0.17
Total IQ	98.8 (13.1)	101.2 (15.3)	2.33 (−1.48, 6.13)	1.88 (−2.19, 5.96)	0.13

Adult ADHD, mean (SD)	(n = 96)	(n = 134)			
ADHD symptom score	8.2 (6.8)	8.4 (6.7)	0.18 (−1.58, 1.95)	0.32 (−1.76, 2.40)	0.05

Abbreviations: ADHD, Attention Deficit Hyperactivity Disorder; WASI II, Wechsler Abbreviated Scale of Intelligence – Version II.

^a^ Adjusted for sex, ethnicity, birthweight z‐score, gestation, duration of breast feeding, parental education and potential selection bias.

For the total cohort, both the unadjusted and adjusted data show minimal evidence of differences in outcome by receipt of ACS. ESs were typically in the range of negligible to small: for continuous outcomes adjusted effect sizes (Cohen’s d) were in the range d = 0.01–0.23 with a median of 0.06; and for dichotomous outcomes, ARRs ranged from 0.78–2.03. There was slightly greater ES variability when considered separately by sex (females: continuous outcomes adjusted d = 0.02–0.41, median 0.15, dichotomous outcomes ARR = 0.18–2.63; males: continuous outcomes adjusted d = 0.01–0.35, median 0.13, dichotomous outcomes ARR = 0.48–6.69) or restricted to EP (continuous outcomes adjusted d = 0.01–0.52, median 0.08, dichotomous outcomes ARR = 0.33–2.98).

In the total cohort, ACS+ status was associated with a doubling of the rate of major depression in the past 12 months (Table 5) and an almost threefold increase amongst EP adults (Table S12). The association was stronger for females than males (Table S7), but the ESs were not significantly different.

There was a small ES of ACS+ associated with lower fasting insulin in females (d = 0.30) and with higher fasting insulin in males (d = 0.20) (Table S5); however, the test of sex by ACS interaction was statistically non‐significant. There was also a small ES of ACS+ associated with lower fasting insulin in EP adults (d = 0.31) (Table S10). In EP adults, ACS had no association with mean BP but the ACS+ group had a nearly threefold higher incidence of elevated BP (systolic ≥130 or diastolic ≥85) (Table S10). ACS+ females had a 60% reduced incidence of metabolic syndrome compared with a modest 20% increase for males, but the test of sex by ACS interaction was statistically non‐significant.

In EP adults, there were moderate ES differences of ACS (d = 0.47–0.52) reflecting greater left ventricular (LV) and arterial elastance (Table S11). ACS+ females also exhibited similar ES differences (d = 0.33–0.41) in LV and arterial elastance (Table S6).

ACS+ status was associated with a reduced incidence of astigmatism >2 dioptres in the better eye in females and an increased incidence of poor visual acuity (LogMAR >0.3) in the better eye in males (Table S6). Further analysis showed these results were not related to retinopathy of prematurity and the tests of sex by steroid effect interactions were non‐significant.

Receipt of ACS was associated with a small (3.4–5.4 point) increase in IQ scores in EP adults (d = 0.32–0.37) (Table S13).

Sensitivity analysis was conducted to test for possible collider bias in the effect size estimates resulting from the inclusion of birthweight z‐score and gestation as covariates; exclusion of these variables produced very similar effect size estimates to those reported above (Table S14).

## 4 COMMENT

### 4.1 Principal findings

In this population‐based VLBW cohort, around half of whom had been exposed to ACS, we found that ACS had minimal impact, positive or negative, on a wide range of physical, cognitive and mental health outcomes at 26–30 years, both for the whole cohort (adjusted ES range d = 0.00–0.22; adjusted RR range 0.78–2.03) and those born at <28‐week gestation. ACS+ status was associated with an increase in depression (ARR 2.03, 95% CI 0.99, 9.88), with a slightly stronger association in EP adults. Sex assignment at birth had negligible relationships to the majority of the results. Our results are similar to those reported at the 30‐year follow‐up of the Auckland randomised controlled trial of ACS.^10^, ^22^, ^23^, ^24^

### 4.2 Strengths of the study

We assessed a prospectively enrolled population‐based sample in young adulthood with good retention, half having received ACS. A range of investigations were undertaken concurrently at one centre. Surfactant was unavailable in New Zealand in 1986, but many elements of modern neonatal intensive care including parenteral nutrition were standard practice.

### 4.3 Limitations of the data

ACS treatment was not randomised and could be biased by several factors. We lack details of the ACS treatment, but common practice at the time was to use betamethasone as a single course. Surviving ACS+ infants tended to be born at a shorter gestation than the ACS‐ group, likely reflecting the effectiveness of antenatal steroid treatment on survival. We recognise that the pattern of findings might differ in other cohorts where survival is different. The cohort was enrolled by birthweight, so small for gestational age infants are overrepresented, but we have separately analysed data for those with gestation <28 weeks (EP) with similar results. Because participant number is relatively small, our study is adequately powered to detect only moderate‐to‐large effect sizes and there is considerable imprecision in our analysis, as reflected in the wide 95% CIs for most ES estimates. In addition, given the large number of effect sizes estimated, it is possible that one or more ESs may appear large simply as a result of chance.

### 4.4 Interpretation

There are several population‐based VLBW or very preterm (VP; <32‐week gestation) cohorts born in the 1970s–1990s from high‐income countries that have been followed up as young adults compared with term‐born controls, most contributing to the Adult Preterm Infant Collaboration (APIC).^34^ A common theme for many health outcomes in these studies is that whilst most VLBW/VP adults are healthy and have physiological measurements in the normal range for age, the mean values for the group are typically less satisfactory than measurements from the control group.^35^, ^36^, ^37^, ^38^ In many APIC cohorts, ACS exposure was well below 50% and often not considered in the analysis of physical health variables.^39^ In a few studies, ACS exposure was reported to have no association with the outcomes of interest.^29^, ^40^, ^41^ To our knowledge, this is the first comprehensive analysis of the relationship of ACS exposure and a range of health outcomes in a VLBW/VP young adult cohort.

Smaller size at birth and an increased risk of abnormal glucose homeostasis in adulthood is one of the key associations of the DOHaD concept.^42^ Hofman reported reduced insulin sensitivity in a convenience sample of 52 children born at <32‐week gestation (68% ACS+), compared with 22 term controls, but ACS status did not affect these results.^43^ Mathai reported increased insulin resistance in 31 surviving preterm‐born adults from the Auckland Steroid Trial (42% ACS+) compared with 21 term adults (48% ACS+). This remained significant after adjusting for confounders including ACS.^44^ One hundred survivors (48% ACS+) of an extremely low birthweight (ELBW;<1000 g) Canadian cohort were followed up at mean age 31.8 years and were 3–4 times more likely to have pre‐diabetes or Type 2 diabetes than 90 term controls, but ACS status was not predictive of this outcome.^40^ Similarly, we found no adverse impact of ACS status on fasting blood insulin, glucose, calculated insulin resistance (HOMA‐IR), glycated haemoglobin or the metabolic syndrome.

A follow‐up of a VLBW hospital cohort at 14 years of age (50% ACS+) reported ACS was associated with increased height and better cognitive function^45^ but higher BP.^46^ An APIC‐related meta‐analysis of 9 VLBW adult cohorts listed ACS rates in each but did not consider ACS in the analysis of BP.^39^ We observed EP ACS+ adults to have a higher incidence of an elevated BP, and this should be explored in future studies.

ACS exposure has been associated with differences in cardiovascular structure and function.^47^, ^48^ Kelly followed a subset of surviving 23–28‐year olds born with birthweight <1850 g included in a neonatal feeding trial.^47^ Sixteen ACS+ survivors were compared with 32 matched ACS‐ preterm controls. Assessments included BP and cardiac MRI. ACS+ adults were reported to have increased aortic arch stiffness compared with the matched ACS‐ controls.^47^ The considerable attrition in this study follow‐up invites caution in interpretation. We measured peripheral artery distensibility and found VLBW young adults had reduced reactive hyperaemia indices compared with term‐born controls, which was unaffected by ACS status.^41^ In EP adults, we observed small‐to‐moderate ES effect of ACS associated with greater LV and arterial elastance, respectively, a load‐independent measure of LV chamber performance and a measure of net arterial load exerted on the LV.^41^ There were similar ES differences in females. These findings suggest future follow‐up is warranted.

Several studies have reported VLBW/VPT adults have decreased expiratory flow variables on spirometry compared with term controls.^36^, ^49^ An individual patient data meta‐analysis of airflow variables in VLBW/VP young adults found ACS exposure (in 41% overall) was associated with increased forced expiratory volume in 1 s (FEV_1_) and forced vital capacity (FVC) z‐scores on both uni‐ and multivariable analysis.^49^ By contrast, we previously reported no difference by ACS status in lung function testing at rest or during exercise.^30^, ^31^

Although a single course of ACS given for expected very preterm delivery is associated with reduced rates of intraventricular haemorrhage,^2^ in animal models adverse effects have been reported in the brain, together with cognitive and behavioural abnormalities.^5^, ^16^, ^18^, ^19^, ^50^ An Australian follow‐up study in childhood found repeat ACS courses were associated with aggressive and hyperactive behaviour.^51^ In a Canadian regional cohort of 84 ELBW survivors (39% ACS+) compared with 90 term controls, ELBW adults had lower odds of substance use disorder and higher odds of non‐substance‐related psychiatric disorders.^52^ ACS+ status increased these odds overall and for generalised anxiety, social phobia and attention‐deficit hyperactivity disorder sub‐type.^52^ Follow‐up at age 19 years of 344 (51% of survivors; 20% ACS+) in the Dutch POPS study (a national cohort born <32 weeks and/or < 1500 g in 1983) found ACS exposure was associated with more internalising behaviour.^53^ A Finnish population registry study of births from 2006 to 2017 (2.3% ACS+) reported at a median 5.8 years that ACS increased the risk of mental or behavioural problems (ACS+ 12%, ACS‐ 6.45%), including in the 45% of ACS+ infants born at term.^54^ Whether these associations persist to young adulthood remains to be determined. We found that receipt of ACS was associated with an increased risk of major depression overall and in those born EP. The effect size was greater in females than males, but there was no sex interaction. In the EP group, receipt of ACS was associated with slightly increased IQ scores, which might reflect the known benefit of ACS in reducing IVH.^2^, ^3^

In a series of publications, Jobe has noted that ACS are now used in a variety of circumstances other than anticipated preterm delivery at 24 to 34 weeks, including very early gestations, late‐preterm deliveries and prior to elective caesarean section. In these circumstances, the benefit to risk ratio of ACS might be different.^7^, ^8^, ^55^, ^56^ In addition, up to 45% of foetuses exposed to ACS for threatened very preterm delivery are born at term.^56^ Because our cohort was both exposed to ACS and born very prematurely, we do not have data that address these concerns directly but our results might provide a stimulus to examine long‐term outcomes from such use.

## 5 CONCLUSIONS

Although adverse outcomes related to ACS have been reported in some studies in adolescence or young adulthood, there is little consistency in the literature and overall reports of harm are few. Despite different study designs, the similarity of findings after exposure to ACS seen in our prospective VLBW cohort and the 30‐year follow‐up of the first RCT of ACS ^10^ is cause for optimism that antenatal steroids have minimal harmful effects when used for threatened very preterm labour, at least by the third decade of life. We did observe an increase in major depression associated with receipt of ACS, which needs confirmation in other studies. Given the emerging data that VLBW/VP adults might be at risk of chronic non‐communicable diseases at an earlier age than their term‐born peers, these cohorts should continue to be followed up as they age. Future studies should report participant numbers exposed to ACS and consider the impact of this variable, alongside environmental factors and lifestyle choices, in the analysis of health outcomes.

## CONFLICT OF INTEREST

The authors declare no conflicts of interest.

## AUTHOR CONTRIBUTION

BAD, SLH, LJH had full access to all the data in the study and take responsibility for the integrity of the data and the accuracy of the data analysis. BAD conceptualized the study and wrote the first draft of the manuscript. LJH performed the statistical analysis. BAD and LJH obtained funding. All authors contributed to the analysis plan and interpretation of the results and reviewed and approved the final manuscript. All authors accept responsibility for the paper as published. BAD is the guarantor.

## Supporting information


Appendix S1

## Data Availability

Author elects to not share data

## References

[1] Liggins Institute . Antenatal corticosteroid clinical practice guidelines panel. antenatal corticosteroids given to women prior to birth to improve fetal, infant, child and adult health: clinical practice guidelines, The University of Auckland; 2015. https://www.ligginstrials.org/ANC_CPG/downloads/Antenatal_Corticosteroid_Clinical_Practice_Guidelines.pdf. Accessed April 13, 2022.

[2] Roberts D , Brown J , Medley N , Dalziel SR . Antenatal corticosteroids for accelerating fetal lung maturation for women at risk of preterm birth. Cochrane Database System Rev. 2017;3:CD004454. 28321847 10.1002/14651858.CD004454.pub3 PMC6464568

[3] McGoldrick E , Stewart F , Parker R , Dalziel SR . Antenatal corticosteroids for accelerating fetal lung maturation for women at risk of preterm birth. Cochrane Database System Rev. 2020;12:CD004454. 33368142 10.1002/14651858.CD004454.pub4 PMC8094626

[4] Yim CL , Tam M , Chan HL , et al. Association of antenatal steroid and risk of retinopathy of prematurity: a systematic review and meta‐analysis. British J Ophthalmol. 2018;102:1336‐1341. 10.1136/bjophthalmol-2017-311576 29632000

[5] Carson R , Monaghan‐Nichols AP , DeFranco DB , Rudine AC . Effects of antenatal glucocorticoids on the developing brain. Steroids. 2016;114:25‐32. 27343976 10.1016/j.steroids.2016.05.012 PMC5052110

[6] Agnew EJ , Ivy JR , Stock SJ , Chapman KE . Glucocorticoids, antenatal corticosteroid therapy and fetal heart maturation. J Mol Endocrinol. 2018;61:R61‐R73. 29720513 10.1530/JME-18-0077 PMC5976079

[7] Jobe AH , Goldenberg RL . Antenatal corticosteroids: an assessment of anticipated benefits and potential risks. Am J Obstet Gynecol. 2018;219:62‐74. 29630886 10.1016/j.ajog.2018.04.007

[8] Jobe AH . Antenatal corticosteroids – a concern for lifelong outcomes. J Pediatr. 2020;217:184‐188. 31606149 10.1016/j.jpeds.2019.09.015

[9] Liggins GC , Howie RN . A controlled trial of antepartum glucocorticoid treatment for prevention of the respiratory distress syndrome in premature infants. Pediatrics. 1972;50:515‐525. 4561295

[10] Dalziel SR , Walker NK , Parag V , et al. Cardiovascular risk factors after antenatal exposure to betamethasone: 30‐year follow‐up of a randomised controlled trial. Lancet. 2005;365:1856‐1862. 15924982 10.1016/S0140-6736(05)66617-2

[11] Liggins GC . Premature delivery of foetal lambs infused with glucocorticoids. J Endocrinol. 1969;45:515‐523. 5366112 10.1677/joe.0.0450515

[12] Barker DJ , Winter PD , Osmond C , Margetts B , Simmonds SJ . Weight in infancy and death from ischaemic heart disease. Lancet. 1989;2(8663):577‐580. 2570282 10.1016/s0140-6736(89)90710-1

[13] Moisiadis VG , Matthews SG . Glucocorticoids and fetal programming part 1: outcomes. Nat Rev Endocrinol. 2014;10:391‐402. 24863382 10.1038/nrendo.2014.73

[14] Harris A , Seckl J . Glucocorticoids, prenatal stress and the programming of disease. Hormones Behav. 2011;59:279‐289. 10.1016/j.yhbeh.2010.06.007 20591431

[15] Seckl JR . Prenatal glucocorticoids and long‐term programming. Eur J Endocrinol. 2004;151:U49‐U62. 15554887 10.1530/eje.0.151u049

[16] Reynolds RM . Glucocorticoid excess and the developmental origins of disease: two decades of testing the hypothesis‐2012 Curt Richter award winner. Psychoneuroendocrinology. 2013;38:1‐11. 22998948 10.1016/j.psyneuen.2012.08.012

[17] Moisiadis VG , Matthews SG . Glucocorticoids and fetal programming part 2: mechanisms. Nat Rev Endocrinol. 2014;10:403‐411. 24863383 10.1038/nrendo.2014.74

[18] McKinlay CJ , Dalziel SR , Harding JE . Antenatal glucocorticoids: where are we after forty years? J Dev Orig Health Dis. 2015;6:127‐142. 25466556 10.1017/S2040174414000579

[19] van der Merwe JL , Sacco A , Toelen J , Deprest J . Long‐term neuropathological and/or neurobehavioral effects of antenatal corticosteroid therapy in animal models: a systematic review. Pediatr Res. 2020;87:1157‐1170. 31822018 10.1038/s41390-019-0712-1

[20] MacArthur BA , Howie RN , Dezoete JA , Elkins J . Cognitive and psychosocial development of 4‐year‐old children whose mothers were treated antenatally with betamethasone. Pediatrics. 1981;68:638‐643. 7031582

[21] MacArthur BA , Howie RN , Dezoete JA , Elkins J . School progress and cognitive development of 6‐year‐old children whose mothers were treated antenatally with betamethasone. Pediatrics. 1982;70:99‐105. 7201129

[22] Dalziel SR , Lim VK , Lambert A , et al. Antenatal exposure to betamethasone: psychological functioning and health related quality of life 31 years after inclusion in randomised controlled trial. BMJ. 2005;331:665‐662. 16143712 10.1136/bmj.38576.494363.E0 PMC1226245

[23] Dalziel SR , Fenwick S , Cundy T , et al. Peak bone mass after exposure to antenatal betamethasone and prematurity: follow‐up of a randomized controlled trial. J Bone Miner Res. 2006;21:1175‐1186. 16869715 10.1359/jbmr.060516

[24] Dalziel SR , Rea HH , Walker NK , et al. Long term effects of antenatal betamethasone on lung function: 30 year follow up of a randomised controlled trial. Thorax. 2006;61:678‐683. 16601084 10.1136/thx.2005.051763 PMC2104681

[25] Smolders‐de Haas H , Neuvel J , Schmand B , Treffers PE , Koppe JG , Hoeks J . Physical development and medical history of children who were treated antenatally with corticosteroids to prevent respiratory distress syndrome: a 10‐ to 12‐year follow‐up. Pediatrics. 1990;86:65‐70. 2193304

[26] Schmand B , Neuvel J , Smolders‐de Haas H , Hoeks J , Treffers PE , Koppe JG . Psychological development of children who were treated antenatally with corticosteroids to prevent respiratory distress syndrome. Pediatrics. 1990;86:58‐64. 2193303

[27] Darlow BA , Horwood LJ , Woodward LJ , et al. The New Zealand 1986 very low birth weight cohort as young adults: mapping the road ahead Study Protocol. BMC Pediatr. 2015;15:90. 26242407 10.1186/s12887-015-0413-9 PMC4526306

[28] Prickett TCR , Darlow BA , Troughton RW , et al. New insights into cardiac and vascular natriuretic peptides: findings from young adults born with very low birth weight. Clin Chem. 2018;64:363‐373. 29097512 10.1373/clinchem.2017.280354

[29] Darlow BA , Martin J , Horwood LJ . Metabolic syndrome in very low birth weight young adults and controls: the NZ 1986 VLBW study. J Pediatr. 2019;206:128‐133. 30545563 10.1016/j.jpeds.2018.10.060

[30] Yang J , Kingsford RA , Horwood LJ , et al. Lung function of adults born at very low birth weight. Pediatrics. 2020;145:e20192359. 31900317 10.1542/peds.2019-2359

[31] Yang J , Epton MJ , Harris SL , et al. Reduced exercise capacity in adults born very low birth weight: a population‐based prospective cohort study. Am J Respir Crit Care Med. 2022;205:88‐98. 34499592 10.1164/rccm.202103-0755OC

[32] Cohen J . Statistical Power Analysis for the Behavioral Sciences. 2nd ed. Lawrence Earlbaum Associates; 1988.

[33] Norton EC , Miller MM , Kleinman LG . Computing adjusted risk ratios and risk differences in Stata. The Stata Journal. 2013;13:492‐509.

[34] Kajantie E , Johnson S , Heinonen K , et al. Common core assessments in follow‐up studies of adults born preterm ‐ recommendation of the adults born preterm international collaboration. Paediatr Perinatal Epidemiol. 2021;35:371‐387. 10.1111/ppe.12694 32990377

[35] Luu TM , Katz SL , Leeson P , Thébaud B , Nuyt AM . Preterm birth: risk factor for early‐onset chronic diseases. Clin Perinatol. 2017;44:305‐314. 26644500 10.1503/cmaj.150450 PMC4938684

[36] Raju TNK , Buist AS , Blaisdell CJ , Moxey‐Mims M , Saigal S . Adults born preterm: a review of general health and system‐specific outcomes. Acta Paediatr. 2017;106:1409‐1437. 28419544 10.1111/apa.13880

[37] Cheong JLY , Haikerwal A , Wark JD , et al. Cardiovascular health profile at age 25 years in adults born extremely preterm or extremely low birthweight. Hypertension. 2020;76:1838‐1846. 33100047 10.1161/HYPERTENSIONAHA.120.15786

[38] Darlow BA , Horwood J , Dhakal B , et al. Biomarkers of ageing in New Zealand VLBW young adults and controls. Pediatr Res. 2021;89:533‐539. 32294664 10.1038/s41390-020-0882-x

[39] Hovi P , Vohr B , Ment LR , et al. Blood pressure in young adults born at very low birth weight: adults born preterm international collaboration. Hypertension. 2016;68:880‐887. 27572149 10.1161/HYPERTENSIONAHA.116.08167

[40] Morrison KM , Ramsingh L , Gunn E , et al. Cardiometabolic health in adults born premature with extremely low birth weight. Pediatrics. 2016;138:e20160515. 27590899 10.1542/peds.2016-0515

[41] Harris S , Bray H , Troughton R , et al. Cardiovascular outcomes in young adulthood in a population‐based very low birth weight cohort. J Pediatr. 2020;225:74‐79. 32553866 10.1016/j.jpeds.2020.06.023

[42] Gluckman PD , Hanson MA , Cooper C , Thornburg KL . Effect of in utero and early‐life conditions on adult health and disease. N Engl J Med. 2008;35:61‐73. 10.1056/NEJMra0708473 PMC3923653 18596274

[43] Hofman PL , Regan F , Jackson WE , et al. Premature birth and later insulin resistance. N Engl J Med. 2004;351:2179‐2186. 15548778 10.1056/NEJMoa042275

[44] Mathai S , Cutfield WS , Derraik JG , et al. Insulin sensitivity and β‐cell function in adults born preterm and their children. Diabetes. 2012;61:2479‐2483. 22596051 10.2337/db11-1672 PMC3447901

[45] Doyle LW , Ford GW , Rickards AL , et al. Antenatal corticosteroids and outcome at 14 years of age in children with birth weight less than 1501 grams. Pediatrics. 2000;106:e2. 10878171 10.1542/peds.106.1.e2

[46] Doyle LW , Ford GW , Davis NM , Callanan C . Antenatal corticosteroid therapy and blood pressure at 14 years of age in preterm children. Clin Sci. 2000;98:137‐142. 10657267

[47] Kelly BA , Lewandowski AJ , Worton SA , et al. Antenatal glucocorticoid exposure and long‐term alterations in aortic function and glucose metabolism. Pediatrics. 2012;129:e1282‐e1290. 22508917 10.1542/peds.2011-3175

[48] Savoy C , Mathewson KJ , Schmidt LA , et al. Exposure to antenatal corticosteroids and reduced respiratory sinus arrhythmia in adult survivors of extremely low birth weight. Int J Neurosci. 2019;129:776‐783. 30633628 10.1080/00207454.2019.1567511

[49] Doyle LW , Andersson S , Bush A , et al. Expiratory airflow in late adolescence and early adulthood in individuals born very preterm or with very low birthweight compared with controls born at term or with normal birthweight: a meta‐analysis of individual participant data. Lancet Resp Med. 2019;7:677‐686. 10.1016/S2213-2600(18)30530-7 31078498

[50] Matthews SG . Antenatal glucocorticoids and programming of the developing CNS. Pediatr Res. 2000;47:291‐300. 10709726 10.1203/00006450-200003000-00003

[51] French NP , Hagan R , Evans SF , Mullan A , Newnham JP . Repeated antenatal corticosteroids: effects on cerebral palsy and childhood behavior. Am J Obstet Gynecol. 2004;190:588‐595. 15041985 10.1016/j.ajog.2003.12.016

[52] Van Lieshout RJ , Boyle MH , Saigal S , Morrison K , Schmidt LA . Mental health of extremely low birth weight survivors in their 30s. Pediatrics. 2015;135:452‐459. 25667243 10.1542/peds.2014-3143

[53] van der Voorn B , Wit JM , van der Pal SM , Rotteveel J , Finken MJ . Antenatal glucocorticoid treatment and polymorphisms of the glucocorticoid and mineralocorticoid receptors are associated with IQ and behavior in young adults born very preterm. J Clin Endocrinol Metabol. 2015;100:500‐507. 10.1210/jc.2014-2843 25406795

[54] Räikkönen K , Gissler M , Kajantie E . Associations between maternal antenatal corticosteroid treatment and mental and behavioral disorders in children. JAMA. 2020;323:1924‐1933. 32427304 10.1001/jama.2020.3937 PMC7237984

[55] Jobe AH , Kemp M , Schmidt A , Takahashi T , Newnham J , Milad M . Antenatal corticosteroids: a reappraisal of the drug formulation and dose. Pediatr Res. 2021;89:318‐325. 33177675 10.1038/s41390-020-01249-w PMC7892336

[56] Jobe AH , Schmidt AF . Chapter for antenatal steroids ‐ treatment drift for a potent therapy with unknown long‐term safety. Seminars in Fetal Neonatal Med. 2021;26:101231. 10.1016/j.siny.2021.101231 33773951

[57] Alberti KG , Zimmet P , Shaw J . Metabolic syndrome—a new worldwide definition. A consensus statement from the International Diabetes Federation. Diabetes Med Med. 2006;23:469‐480. 10.1111/j.1464-5491.2006.01858.x 16681555

