# Supplementary material for: Little evidence for long‐term harm from antenatal corticosteroids in a population‐based very low birthweight young adult cohort
Source: Paediatr Perinat Epidemiol. 2022 May 16;36(5):631–9. doi: 10.1111/ppe.12886 (PMC9545416; doi:10.1111/ppe.12886)
Supplement: Supplementary file 1 — Appendix S1 [file PPE-36-631-s001.docx]

**Supplemental material for:**

Little evidence for long term harm from antenatal corticosteroids in a population-based very low birthweight young adult cohort

Darlow BA, Harris SL, Horwood LJ

eTable 1 Summary of investigations over two days at age 26-30 years for VLBW cohort (n=229) and term born controls (n=100)

eTable 2 Demographics and perinatal data of the VLBW cohort who were assessed and not assessed on (a) clinical assessments and (b) interview assessment

eTable 3 Demographics and perinatal data of the VLBW cohort by receipt of antenatal corticosteroids (ACS) – interview assessment sample

eTable 4 Adult growth outcomes by receipt antenatal corticosteroids (ACS) and sex

eTable 5 Biomedical and metabolic indices by receipt of antenatal corticosteroids (ACS) and sex

eTable 6 Visual and cardiovascular outcomes by receipt of antenatal corticosteroids (ACS) and sex

eTable 7 Adult mental health, substance use and antisocial behaviour outcomes by receipt of antenatal corticosteroids (ACS) and sex

eTable 8 Adult IQ and ADHD by receipt of antenatal corticosteroids (ACS) and sex

eTable 9 Adult growth outcomes by receipt of antenatal corticosteroids (ACS) amongst extremely preterm (<28 weeks) infants

eTable 10 Biomedical and metabolic indices by receipt of antenatal corticosteroids (ACS) amongst extremely preterm (<28 weeks) infants

eTable 11 Visual and cardiovascular outcomes by receipt of antenatal corticosteroids (ACS) amongst extremely preterm (<28 weeks) infants

eTable 12 Adult mental health, substance use and antisocial behaviour outcomes by antenatal corticosteroids (ACS) amongst extremely preterm (<28 weeks) infants

eTable 13 Adult IQ and ADHD by receipt of antenatal corticosteroids (ACS) amongst extremely preterm (<28 weeks) infants

eTable 14 Adjusted effect size estimates for antenatal corticosteroids (ACS) from analyses excluding gestation, birthweight z-score as covariates.

eFigure 1 New Zealand VLBW Follow-up Study cohort flow chart

eTable 1. Summary of outcome measures assessed in New Zealand VLBW cohort at age 26-30 years and used in analysis of relationship of antenatal corticosteroids to adult health and welfare

| Growth: Height (Harpenden stadiometer), weight, BMI, waist-hip ratio, body fat (bioelectrical impedence)^a^  Blood tests: (Following an overnight fast) plasma glucose, (free) insulin (insulin resistance was calculated from the homeostasis model assessment of insulin resistance (HOMA-IR), glycated haemoglobin (HbA1c), lipid screen (cholesterol, triglycerides, high-density lipoprotein cholesterol, total cholesterol)^a^  Cardiovascular: Blood pressure (BP), electrocardiogram, ultrasound echocardiogram, peripheral artery tonometry (reactive hyperaemic index: RHI) using the EndoPAT system^b^  Vision: Distance visual acuity (VA), contrast sensitivity, auto-refraction^c^  Cognitive functioning: Wechsler Abbreviated Scale of Intelligence – Second Edition (WASI-II)  Questionnaire (250 VLBW cohort): Mental health, including structured diagnostic tools to assess substance abuse; mood disorders/depression; anxiety disorders.  In addition, attention-deficit/hyperactivity disorder (ADHD) symptoms were assessed at 22-23 years at a face-to-face interview using the Barkley Adult ADHD Rating Scale-IV^d^ |
| --- |

Full details of investigations previously reported in: ^a^Darlow et al *J Pediatr* 2019;206:128-33; ^b^Harris et al *J Pediatr* 2020;225:74-9; ^c^Darlow et al *Brit J Ophthalmol* 2018;102:1041-6; ^d^Darlow et al *Pediatrics* 2013; 132: e1521-1528

eTable 2. Demographics and perinatal data of the VLBW cohort who were assessed and not assessed on (a) clinical assessments and (b) interview assessment

| Measure | Clinical Assessment | | |  | Interview Assessment | | |
| --- | --- | --- | --- | --- | --- | --- | --- |
|  | Assessed  (N=229) | Not  assessed^e^ (N=94) | Difference  (95% CI) |  | Assessed  (N=250) | Not assessed^f^ (N=73) | Difference  (95% CI) |
| Male, % | 44.5 | 53.2 | -8.7 (-3.3, 20.6) |  | 42.8 | 61.6 | -18.8 (-31.6, -6.1) |
| Māori/Pacific Island, % | 31.0 | 34.0 | -3.0 (-14.3, 8.3) |  | 30.8 | 35.6 | -4.8 (-17.2, 7.6) |
| Gestation (weeks), mean (sd) | 29.2 (2.5) | 29.3 (2.4) | -0.07 (-0.67, 0.53) |  | 29.2 (2.5) | 29.5 (2.4) | -0.33 (-0.98, 0.31) |
| <28 weeks gestation,% | 24.9 | 24.5 | 0.4 (-9.9, 10.8) |  | 26.0 | 20.6 | 5.4 (-5.3, 16.2) |
| Birthweight (g), mean(sd) | 1133 (237) | 1192 (230) | -59.2 (-115.7, -2.6) |  | 1134 (236) | 1206 (228) | -72.8 (-134.1, -11.5) |
| <1000g, % | 28.0 | 21.3 | 6.7 (-3.4, 16.8) |  | 27.2 | 21.9 | 5.3 (-5.7, 16.3) |
| Small for gestational age^a^, % | 31.4 | 22.3 | 9.1 (-1.2, 19.5) |  | 30.0 | 24.7 | 5.3 (-6.1, 16.8) |
| Antenatal corticosteroids, % | 56.3 | 59.6 | -3.2 (-15.1, 8.6) |  | 56.4 | 60.3 | -3.9 (-16.7, 8.9) |
| Respiratory distress syndrome, % | 54.6 | 60.6 | -6.1 (-17.9, 5.7) |  | 56.4 | 56.2 | 0.2 (-12.7, 13.2) |
| Bronchopulmonary dysplasia^b^, % | 20.1 | 23.4 | -3.3 (-13.3, 6.7) |  | 20.4 | 23.2 | -2.3 (-13.8, 0.8) |
| Retinopathy of prematurity, % | 19.7 | 19.2 | 0.5 (-9.0, 10.0) |  | 21.6 | 12.3 | 9.3 (0.2, 18.4) |
| Duration breast feeding (months), mean (sd) | 4.4 (5.8) | 5.1 (7.9) | -0.69 (-2.38, 1.00) |  | 4.5 (5.9) | 5.2 (8.3) | -0.67 (-2.56, 1.22) |
| Parental education^c^, mean (sd) | 2.0 (0.8) | 2.0 (0.8) | 0.03 (-0.19, 0.24) |  | 2.1 (0.8) | 2.0 (0.9) | 0.03 (-0.21, 0.27) |
| Any neurosensory disability (age 7-8 years), % | 23.0 | 31.1 | -8.1 (-20.0, 3.8) |  | 24.8 | 25.9 | -1.1 (-14.0, 11.8) |
| Mod/severe disability^d^ (age 7-8 years), % | 5.9 | 18.9 | -13.6 (-22.5, -3.6) |  | 8.7 | 11.1 | -2.4 (-11.5, 6.7) |

^a^ birthweight <10% centile ^b^ oxygen requirement at 36 weeks post-menstrual age ^c^ Parental education scored in 3 levels (no formal qualifications/high school qualifications/tertiary qualifications) based on highest educational attainment of either parent
^d^ Moderate or severe disability at 7-8 years of age was defined as cerebral palsy in non-ambulant children or in ambulant children causing considerable limitation of movement, or bilateral sensorineural deafness requiring hearing aids, or bilateral blindness, or an IQ score of >2 SD below the test mean (<70) on the Revised Wechsler Intelligence Scale for Children (WISC-R).(Darlow BA et al. *J Paediatr Child Health* 1997;33:47-51) ^e^ Includes 73 with no follow-up (35 not able to be contacted [13 known to be overseas], 38 contacted but declined); and 21 not assessed in Christchurch but who consented to an interview.
^f^ Includes 73 with no follow-up (35 not able to be contacted [13 known to be overseas], 38 contacted but declined)

eTable 3. Demographics and perinatal data of the VLBW cohort by receipt of antenatal corticosteroids (ACS) – interview assessment sample

|  | Receipt of ACS | | Difference  (95% CI) |
| --- | --- | --- | --- |
| Measure | No (N=109) | Yes (N=141) |  |
| Age at assessment (yrs), mean (sd) | 28.5 (1.1) | 28.5 (1.1) | -0.03 (-0.31, 0.25) |
| Male, % | 44.9 | 41.1 | -3.8 (-16.2, 8.6) |
| Māori/Pacific Island, % | 33.9 | 28.4 | -5.6 (-17.2, 6.0) |
| Gestation (weeks), mean (sd) | 29.8 (2.9) | 28.7 (2.0) | -1.13 (-1.74, -0.51) |
| <28 weeks gestation, % | 22.9 | 28.4 | 5.4 (-5.4, 16.3) |
| Birthweight (g), mean (sd) | 1141 (225) | 1128 (245) | -13.3 (-72.7, 46.2) |
| <1000g, % | 24.8 | 29.1 | 4.3 (-6.7, 15.4) |
| Birthweight z-score, mean (sd) | -1.1 (1.3) | -0.5 (1.0) | 0.58 (0.28, 0.88) |
| Small for gestational age, % | 39.5 | 22.7 | -16.8 (-28.2, -5.3) |
| Respiratory distress syndrome, % | 55.1 | 57.4 | 2.4 (-10.0, 14.8) |
| Bronchopulmonary dysplasia, % | 25.7 | 16.3 | -9.4 (-19.6, 0.8) |
| Apgar score (5 min), median (IQR) | 8.0 (6.0-9.0) | 9.0 (7.0-9.0) | 1.0 (0.41-1.59) |
| Retinopathy of prematurity, % | 17.4 | 24.8 | 7.4 (-3.7, 17.5) |
| Necrotising enterocolitis, % | 13.8 | 9.9 | -3.8 (-12.0, 4.3) |
| Maternal pre-eclamptic toxaemia, % | 27.5 | 21.3 | -6.3 (-17.0, 4.5) |
| Born in a Level III centre, % | 66.1 | 76.6 | 10.5 (-0.8, 21.9) |
| Maternal smoking during pregnancy, % | 40.4 | 36.8 | -3.5 (-15.7, 8.7) |
| Duration breast feeding (mths), mean (sd) | 4.9 (5.7) | 4.2 (6.0) | -0.74 (-2.25, 0.76) |
| Maternal age at childbirth, mean (sd) | 25.9 (5.4) | 26.0 (4.9) | 0.03 (-1.28, 1.34) |
| Parental education^a^, mean (sd) | 2.0 (0.8) | 2.2 (0.8) | 0.20 (-0.00, 0.40) |

IQR: interquartile range.

^a^ Parental education scored in 3 levels (no formal qualifications/high school qualifications/tertiary qualifications) based on highest educational attainment of either parent

eTable 4. Adult growth outcomes by receipt antenatal corticosteroids (ACS) and sex

|  | Receipt of ACS | |  | Mean difference (95%CI) | |  |
| --- | --- | --- | --- | --- | --- | --- |
| Measure | No | Yes |  | Unadjusted | Adjusted^a^ | Cohen’s d^a^ |
| Females | (N=53) | (N=74) |  |  |  |  |
| Mean height, cm | 161.5 (5.7) | 161.2 (6.5) |  | -0.24 (-2.44, 1.96) | -1.11 (-3.41, 1.20) | 0.18 |
| Weight, kg | 73.1 (20.0) | 69.2 (18.7) |  | -3.85 (-10.78, 3.09) | -2.94 (-10.12, 4.23) | 0.15 |
| BMI, kg/m^2^ | 27.8 (6.8) | 26.5 (6.8) |  | -1.30 (-3.74, 1.15) | -0.70 (-3.23, 1.83) | 0.10 |
| Waist circumference | 83.0 (15.1) | 80.7 (14.6) |  | -2.25 (-7.52, 3.02) | -1.97 (-7.31, 3.38) | 0.13 |
| Hip circumference, cm | 101.9 (13.5) | 100.3 (13.8) |  | -1.59 (-6.47, 3.29) | -1.06 (-6.17, 4.06) | 0.08 |
| Waist/hip ratio | 0.81 (0.09) | 0.80 (0.08) |  | -0.01 (-0.04, 0.02) | -0.01 (-0.04, 0.01) | 0.12 |
| Body fat percentage, % | 34.1 (9.2) | 31.9 (10.1) |  | -2.25 (-5.85, 1.36) | -1.56 (-5.25, 2.13) | 0.16 |
| Fat mass, kg | 26.6 (15.0) | 23.7 (13.7) |  | -2.93 (-8.20, 2.34) | -2.07 (-7.54, 3.39) | 0.15 |
| Fat free mass, kg | 46.4 (5.9) | 45.1 (5.3) |  | -1.29 (-3.34, 0.76) | -1.14 (-3.31, 1.02) | 0.21 |
| Total body water, kg | 33.9 (4.3) | 33.0 (3.9) |  | -0.94 (-2.44, 0.56) | -0.83 (-2.41, 0.76) | 0.20 |
| Males | (N=47) | (N=55) |  |  |  |  |
| Mean height, cm | 173.5 (6.2) | 175.4 (5.4) |  | 1.97 (-0.32, 4.26) | 0.50 (-2.02, 3.03) | 0.09 |
| Weight, kg | 78.0 (15.7) | 83.6 (19.3) |  | 5.56 (-1.49, 12.62) | 2.65 (-4.88, 10.17) | 0.15 |
| Body mass index, kg/m^2^ | 25.8 (4.9) | 27.1 (6.1) |  | 1.33 (-0.86, 3.52) | 0.82 (-1.55, 3.19) | 0.15 |
| Waist circumference | 86.6 (14.1) | 88.9 (14.5) |  | 2.37 (-3.26, 8.01) | 1.14 (-5.08 7.36) | 0.08 |
| Hip circumference, cm | 97.4 (9.2) | 101.6 (10.6) |  | 4.23 (0.29, 8.17) | 2.64 (-1.74, 7.02) | 0.26 |
| Waist/hip ratio | 0.88 (0.08) | 0.87 (0.07) |  | -0.01 (-0.04, 0.02) | -0.01 (-0.04, 0.01) | 0.13 |
| Body fat percentage, % | 21.5 (8.3) | 22.3 (8.8) |  | 0.81 (-2.60, 4.21) | -0.39 (-4.13, 3.36) | 0.05 |
| Fat mass, kg | 17.9 (10.7) | 20.5 (14.1) |  | 2.56 (-2.47, 7.59) | 0.76 (-4.72, 6.23) | 0.06 |
| Fat free mass, kg | 60.1 (7.4) | 63.6 (7.0) |  | 3.47 (0.62, 6.33) | 2.46 (-0.63, 5.54) | 0.33 |
| Total body water, kg | 44.0 (5.4) | 46.6 (5.1) |  | 2.54 (0.45, 4.63) | 1.80 (-0.46, 4.05) | 0.34 |

^a^ Adjusted for ethnicity, birthweight z-score, gestation, parental education, duration of breast feeding and potential selection bias

eTable 5. Biomedical and metabolic indices by receipt of antenatal corticosteroids (ACS) and sex

1. Females

|  | Receipt of ACS | |  | Mean difference/RR (95% CI) | |  |
| --- | --- | --- | --- | --- | --- | --- |
| Measure | No | Yes |  | Unadjusted | Adjusted^a^ | Cohen’s d^a^ |
| Bloods, mean (sd) | (N=53) | (N=72) |  |  |  |  |
| Fasting blood insulin (pmol/L) | 83.0 (52.8) | 65.9 (38.4) |  | -17.14 (-33.22, -1.06) | -15.15 (-30.94, 0.65) | 0.30 |
| Fasting blood glucose (mmol/L) | 4.9 (0.5) | 5.0 (0.5) |  | 0.04 (-0.14, 0.23) | 0.03 (-0.17, 0.24) | 0.06 |
| Haemoglobin A1c (mmol/mol) | 31.5 (4.0) | 32.5 (5.2) |  | 0.96 (-0.72, 2.65) | 1.42 (-0.34, 3.19) | 0.30 |
| HOMA-IR | 2.6 (1.7) | 2.1 (1.3) |  | -0.49 (-1.03, 0.05) | -0.45 (-0.97, 0.08) | 0.30 |
| Blood pressure, mean (sd) | (N=53) | (N=74) |  |  |  |  |
| Systolic BP (mm Hg) | 109.5 (11.4) | 108.4 (11.1) |  | -1.10 (-5.09, 2.89) | -1.87 (-6.24, 2.49) | 0.17 |
| Diastolic BP (mm Hg) | 70.7 (8.3) | 71.3 (8.1) |  | 0.58 (-2.35, 3.51) | 0.20 (-3.02, 3.42) | 0.02 |
| Metabolic syndrome^b^, % | (N=53) | (N=73) |  | RR (95%CI) | ARR (95%CI) |  |
| Elevated waist circumference | 47.2 | 45.2 |  | 0.96 (0.65, 1.40) | 0.96 (0.65, 1.43) |  |
| Elevated triglycerides | 20.8 | 9.7 |  | 0.47 (0.19, 1.13) | 0.39 (0.15, 1.02) |  |
| Reduced HDL-C | 47.2 | 45.2 |  | 0.93 (0.64, 1.36) | 1.02 (0.69, 1.51) |  |
| Elevated BP | 5.7 | 9.6 |  | 1.69 (0.46, 6.25) | 1.70 (0.38, 7.66) |  |
| Elevated fasting glucose | 9.4 | 12.5 |  | 1.32 (0.47, 3.73) | 1.58 (0.55, 4.51) |  |
| Metabolic syndrome | 18.9 | 8.3 |  | 0.44 (0.17, 1.14) | 0.39 (0.15, 1.03) |  |

1. Males

|  | Receipt of ACS | |  | Mean difference/RR (95% CI) | |  |
| --- | --- | --- | --- | --- | --- | --- |
| Measure | No | Yes |  | Unadjusted | Adjusted^a^ | Cohen’s d^a^ |
| Bloods, mean (sd) | (N=46) | (N=53) |  |  |  |  |
| Fasting blood insulin (pmol/L) | 68.2 (46.8) | 79.1 (83.9) |  | 10.91 (-17.07, 38.90) | 13.61 (-18.26, 45.47) | 0.20 |
| Fasting blood glucose (mmol/L) | 5.1 (0.5) | 5.2 (0.5) |  | 0.07 (-0.12, 0.26) | 0.05 (-0.17, 0.26) | 0.10 |
| Haemoglobin A1c (mmol/mol) | 31.6 (4.7) | 31.8 (3.4) |  | 0.23 (-1.42, 1.88) | 0.68 (-1.03, 2.40) | 0.17 |
| HOMA-IR | 2.2 (1.6) | 2.6 (2.8) |  | 0.38 (-0.54, 1.31) | 0.46 (-0.59, 1.51) | 0.20 |
| Blood pressure, mean (sd) | (N=47) | (N=55) |  |  |  |  |
| Systolic BP (mm Hg) | 119.9 (11.4) | 120.1 (11.9) |  | 0.25 (-4.35, 4.85) | -0.19 (-5.55, 5.16) | 0.02 |
| Diastolic BP (mm Hg) | 77.8 (10.0) | 77.4 (9.1) |  | -0.436 (-4.12, 3.39) | -0.34 (-4.34, 3.65) | 0.04 |
| Metabolic syndrome^b^, % | (N=46) | (N=53) |  | RR (95%CI) | ARR (95%CI) |  |
| Elevated waist circumference | 26.1 | 32.1 |  | 1.23 (0.66, 2.30) | 1.38 (0.70, 2.74) |  |
| Elevated triglycerides | 28.3 | 28.3 |  | 1.00 (0.53, 1.88) | 1.29 (0.63, 2.64) |  |
| Reduced HDL-C | 23.9 | 37.7 |  | 1.58 (0.85, 2.94) | 1.69 (0.88, 3.26) |  |
| Elevated BP | 34.8 | 26.4 |  | 0.76 (0.42, 1.38) | 0.81 (0.40, 1.62) |  |
| Elevated fasting glucose | 13.0 | 26.4 |  | 2.02 (0.85, 4.83) | 1.50 (0.56, 4.06) |  |
| Metabolic syndrome | 21.7 | 22.6 |  | 1.04 (0.50, 2.18) | 1.21 (0.56, 2.61) |  |

HOMA-IR: homeostatic model assessment for insulin resistance, BP: blood pressure, HDL–C: high density lipoprotein cholesterol  ^a^ Adjusted for ethnicity, birthweight z-score, gestation, duration of breast feeding, parental education and potential selection bias
^b^ Symptoms of metabolic syndrome and elevated waist circumference defined according to International Diabetes Federation criteria (Alberti KG et al. Diabet Med 2006;23:469-80)

eTable 6. Visual and cardiovascular outcomes by receipt of antenatal corticosteroids (ACS) and sex

(a) Females

|  | Receipt of ACS | |  | Mean difference/RR (95% CI) | |  |
| --- | --- | --- | --- | --- | --- | --- |
| Measure | No | Yes |  | Unadjusted | Adjusted^a^ | Cohen’s d^a^ |
| Visual outcomes, % | (N=53) | (N=74) |  | RR (95%CI) | ARR (95%CI) |  |
| Visual acuity (logMAR) >0.3 better eye | 7.6 | 6.8 |  | 0.90 (0.25, 3.18) | 0.80 (0.23, 2.71) |  |
| Myopia >2.0 D better eye | 17.3 | 16.4 |  | 0.95 (0.43, 2.09) | 1.03 (0.44, 2.43) |  |
| Hypermetropia >2.0 D better eye | 0.0 | 0.0 |  | - | - |  |
| Astigmatism >2.0 D better eye | 9.6 | 2.7 |  | 0.28 (0.06, 1.41) | 0.18 (0.04, 0.80) |  |
| Moderate visual impairment (any of the above) | 24.5 | 21.6 |  | 0.88 (0.46, 1.67) | 0.87 (0.43, 1.76) |  |
| Heart structure and function, mean(sd) | (N=52) | (N=74) |  | Mean diff (95%CI) | Mean diff (95%CI) |  |
| LV mass - indexed BSA (g/m^2^) | 86.9 (19.1) | 81.0 (17.2) |  | -5.93 (-12.23, 0.36) | -4.62 (-11.57, 2.33) | 0.26 |
| LVEDV - indexed BSA (mL/m^2^) | 55.4 (8.2) | 55.0 (10.2) |  | -0.38 (-3.77, 3.01) | -0.95 (-4.71, 2.81) | 0.10 |
| LVESV - indexed BSA (mL/ m^2^) | 19.2 (3.5) | 19.2 (4.2) |  | 0.01 (-1.42, 1.39) | 0.04 (-1.54, 1.61) | 0.01 |
| LV elastance (mm Hg/mL) | 3.46 (0.70) | 3.73 (1.0) |  | 0.27 (-0.05, 0.59) | 0.29 (-0.07, 0.65) | 0.33 |
| Arterial elastance (mm Hg/mL) | 1.82 (0.32) | 1.96 (0.47) |  | 0.14 (-0.01, 0.28) | 0.17 (0.00, 0.33) | 0.41 |
| RHI^b^ | 1.80 (0.59) | 1.95 (0.57) |  | 0.15 (-0.07, 0.38) | 0.07 (-0.17. 0.31) | 0.12 |
| Cardiac output - indexed BSA (L/min) | 2.59 (0.55) | 2.60 (0.67) |  | 0.01 (-0.21, 0.23) | 0.01 (-0.23, 0.26) | 0.02 |

(b) Males

|  | | Receipt of ACS | |  | Mean difference/RR (95% CI) | |  |
| --- | --- | --- | --- | --- | --- | --- | --- |
| Measure | No | | Yes |  | Unadjusted | Adjusted^a^ | Cohen’s d^a^ |
| Visual outcomes, % | | (N=47) | (N=55) |  | RR (95%CI) | ARR (95%CI) |  |
| Visual acuity (logMAR) >0.3 better eye | | 2.1 | 12.7 |  | 5.98 (0.76, 46.88) | 6.69 (0.88, 50.57) |  |
| Myopia >2.0 D better eye | | 12.7 | 5.7 |  | 0.44 (0.12, 1.68) | 0.61 (0.12, 3.07) |  |
| Hypermetropia >2.0 D better eye | | 0.0 | 7.6 |  | - | - |  |
| Astigmatism >2.0 D better eye | | 6.4 | 7.6 |  | 1.18 (0.28, 5.01) | 1.35 (0.39, 4.65) |  |
| Moderate visual impairment (any of the above) | | 19.2 | 25.5 |  | 1.33 (0.63, 2.79) | 1.43 (0.66, 3.10) |  |
| Heart structure and function, mean(sd) | | (N=47) | (N=55) |  | Mean diff (95%CI) | Mean diff (95%CI) |  |
| LV mass - indexed BSA (g/m^2^) | | 94.2 (15.6) | 100.0 (20.2) |  | 5.76 (-1.41, 12.93) | 4.42 (-3.95, 12.80) | 0.24 |
| LVEDV - indexed BSA (mL/m^2^) | | 63.2 (11.8) | 61.5 (11.1) |  | -1.74 (-6.25, 2.76) | -2.24 (-7.57, 3.10) | 0.20 |
| LVESV - indexed BSA (mL/ m^2^) | | 23.4 (5.8) | 22.4 (4.6) |  | -0.99 (-3.04, 1.06) | -1.44 (-3.82, 0.94) | 0.28 |
| LV elastance (mm Hg/mL) | | 3.15 (0.88) | 3.03 (0.69) |  | -0.12 (-0.42, 0.19) | 0.01 (-0.33, 0.35) | 0.01 |
| Arterial elastance (mm Hg/mL) | | 1.81 (0.39) | 1.73 (0.35) |  | -0.08 (-0.23, 0.06) | -0.05 (-0.21, 0.12) | 0.13 |
| Reactive hyperaemic index (RHI)^b^ | | 2.02 (0.64) | 1.85 (0.48) |  | -0.17 (-0.41, 0.07) | -0.20 (-0.46, 0.06) | 0.35 |
| Cardiac output - indexed BSA (L/min) | | 2.79 (0.70) | 2.76 (0.58) |  | -0.03 (-0.29, 0.22) | -0.06 (-0.36, 0.24) | 0.09 |

logMAR: log of minimum angle of resolution, D: dioptres, LV: left ventricular, BSA: body surface area, LVEDV: LV end-diastolic volume, LVESV: LV end-systolic volume.

^a^ Adjusted for ethnicity, birthweight z-score, gestation, duration of breast feeding, parental education and potential selection bias
^b^ Sample sizes for RHI – Females no ACS (N=47), ACS (N=64); Males no ACS (N=42) ACS (N=46)

eTable 7. Adult mental health, substance use and antisocial behaviour outcomes by receipt of antenatal corticosteroids (ACS) and sex

1. Females

| Measure | Receipt of ACS | | Unadjusted  RR (95% CI) | Adjusted RR (95% CI)^a^ |
| --- | --- | --- | --- | --- |
|  | No  (N=60) | Yes  (N=83) |  |  |
| Mental health (past 12 months), % |  |  |  |  |
| Major depression | 8.3 | 18.1 | 2.17 (0.83, 5.64) | 2.63 (0.88, 7.83) |
| Suicidal ideation | 6.7 | 8.4 | 1.26 (0.39, 4.13) | 2.04 (0.47, 8.92) |
| Anxiety disorder | 30.0 | 34.9 | 1.16 (0.72, 1.89) | 1.31 (0.76, 2.26) |
| Any of the above | 35.0 | 42.2 | 1.20 (0.79, 1.85) | 1.26 (0.77, 2.04) |
| Substance use/antisocial behaviour, % |  |  |  |  |
| Daily smoker | 30.0 | 22.9 | 0.76 (0.44, 1.33) | 0.93 (0.52, 1.67) |
| Regular (weekly) binge drinking | 13.3 | 12.1 | 0.90 (0.8, 2.16) | 1.70 (0.66, 4.37) |
| Daily cannabis use | 3.3 | 4.8 | 1.44 (0.27, 7.64) | 2.27 (0.64, 8.04) |
| History of adult offending (>18 yrs) | 16.7 | 10.8 | 0.65 (0.28, 1.50) | 0.78 (0.35, 1.72) |

1. Males

| Measure | Receipt of ACS | | Unadjusted  RR (95% CI) | Adjusted RR (95% CI)^a^ |
| --- | --- | --- | --- | --- |
|  | No  (N=49) | Yes  (N=58) |  |  |
| Mental health (past 12 months), % |  |  |  |  |
| Major depression | 10.2 | 14.0 | 1.38 (0.48, 3.93) | 1.58 (0.64, 3.91) |
| Suicidal ideation | 8.2 | 3.5 | 0.42 (0.08, 2.21) | 0.48 (0.13,1.83) |
| Anxiety disorder | 20.4 | 17.2 | 0.84 (0.38, 1.86) | 1.01 (0.42, 2.41) |
| Any of the above | 28.6 | 31.0 | 1.09 (0.60, 1.95) | 1.07 (0.57, 2.02) |
| Substance use/antisocial behaviour, % |  |  |  |  |
| Daily smoker | 36.7 | 34.5 | 0.94 (0.56, 1.56) | 1.35 (0.77, 2.39) |
| Regular (weekly) binge drinking | 24.5 | 15.2 | 0.63 (0.29, 1.38) | 0.77 (0.32, 1.84) |
| Daily cannabis use | 12.2 | 12.1 | 0.98 (0.35, 2.74) | 1.00 (0.34, 2.94) |
| History of adult offending (>18 yrs) | 42.9 | 27.6 | 0.64 (0.38, 1.09) | 0.90 (0.46, 1.76) |

^a^ Adjusted for ethnicity, birthweight z-score, gestation, parental education and potential selection bias

eTable 8. Adult IQ and attention deficit hyperactivity disorder (ADHD) by receipt of antenatal corticosteroids (ACS) and sex

| Measure | Receipt of ACS | |  | Mean difference (95% CI) | |  |
| --- | --- | --- | --- | --- | --- | --- |
|  | No | Yes |  | Unadjusted | Adjusted^a^ | Cohen’s d^a^ |
| Females |  |  |  |  |  |  |
| WASI II IQ scores, mean (sd) | (N=53) | (N=74) |  |  |  |  |
| Verbal IQ | 98.6 (12.0) | 102.5 (15.2) |  | 3.84 (-1.12, 8.80) | 2.13 (-2.20, 6.47) | 0.15 |
| Perceptual IQ | 98.0 (13.1) | 99.5 (13.9) |  | 1.45 (3.42, 6.33) | 2.90 (-1.84,7.64) | 0.21 |
| Total IQ | 98.2 (12.2) | 101.2 (14.3) |  | 3.00 (-1.83, 7.84) | 2.78 (-1.56, 7.12) | 0.21 |
| Adult ADHD, mean (sd) | (N=52) | (N=74) |  |  |  |  |
| ADHD symptom score | 8.3 (7.2) | 9.3 (7.0) |  | 1.00 (-1.53, 3.55) | 0.56 (-2.41, 3.53) | 0.08 |
| Males |  |  |  |  |  |  |
| WASI II IQ scores, mean (sd) | (N=47) | (N=54) |  |  |  |  |
| Verbal IQ | 99.8 (11.5) | 99.9 (15.7) |  | 0.04 (-5.44, 5.52) | -0.98 (-7.36, 5.41) | 0.07 |
| Perceptual IQ | 98.9 (16.7) | 102.1 (17.4) |  | 3.19 (-3.59, 9.99) | 2.40 (-6.34, 11.13) | 0.14 |
| Total IQ | 99.5 (14.1) | 101.1 (16.8) |  | 1.56 (-4.63, 7.75) | 0.56 (-7.14, 8.27) | 0.04 |
| Adult ADHD, mean (sd) | (N=44) | (N=60) |  |  |  |  |
| ADHD symptom score | 8.2 (6.3) | 7.4 (6.1) |  | -0.84 (-3.28, 1.59) | 0.08 (-2.84, 3.00) | 0.01 |

WASI II: Wechsler Abbreviated Scale of Intelligence – Version II, ADHD: Attention Deficit Hyperactivity Disorder
^a^ Adjusted for ethnicity, birthweight z-score, gestation, duration of breast feeding, parental education and potential selection bias

eTable 9. Adult growth outcomes by receipt of antenatal corticosteroids (ACS) amongst extremely preterm (<28 weeks) infants

|  | Receipt of ACS | |  | Mean difference (95%CI) | |  |
| --- | --- | --- | --- | --- | --- | --- |
| Measure | No  (N=22) | Yes  (N=35) |  | Unadjusted | Adjusted^a^ | Cohen’s d^a^ |
| Mean height, cm | 168.9 (7.6) | 166.0 (8.0) |  | -2.70 (-7.04, 1.64) | -1.78 (-5.22, 1.66) | 0.23 |
| Weight, kg | 79.6 (18.8) | 74.0 (22.6) |  | -5.54 (-17.30, 6.23) | -2.75 (-13.67, 8.17) | 0.13 |
| BMI, kg/m^2^ | 27.9 (6.6) | 26.7 (7.5) |  | -1.15 (-5.14, 2.84) | -0.46 (-4.40, 3.49) | 0.06 |
| Waist circumference, cm | 89.3 (19.1) | 86.0 (17.1) |  | -3.37 (-13.12, 6.38) | -1.24 (-10.92, 8.43) | 0.07 |
| Hip circumference, cm | 102.6 (12.3) | 100.7 (15.1) |  | -1.94 (-9.62, 5.75) | -1.35 (-9.45, 6.74) | 0.10 |
| Waist/hip ratio | 0.86 (0.10) | 0.85 (0.11) |  | -0.01 (-0.07, 0.05) | -0.00 (-0.06, 0.06) | 0.00 |
| Body fat percentage, % | 30.6 (10.9) | 28.9 (11.1) |  | -1.73 (-7.90, 4.44) | -0.75 (-6.00, 4.50) | 0.07 |
| Fat mass, kg | 25.7 (13.9) | 23.4 (15.1) |  | -2.31 (-10.51, 5.90) | -0.54 (-8.17, 7.08) | 0.04 |
| Fat free mass, kg | 53.9 (9.2) | 51.6 (11.3) |  | -2.27 (-8.17, 3.63) | -0.13 (-4.30, 4.05) | 0.01 |
| Total body water, kg | 39.4 (6.7) | 37.8 (8.3) |  | -1.66 (-5.98, 2.65) | -0.09 (-3.15, 2.96) | 0.01 |

^a^ Adjusted for sex, ethnicity, birthweight z-score, parental education, duration of breast feeding and potential selection bias

eTable 10. Biomedical and metabolic indices by receipt of antenatal corticosteroids (ACS) amongst extremely preterm (<28 weeks) infants

|  | Receipt of ACS | |  | Mean difference/RR (95% CI) | |  |
| --- | --- | --- | --- | --- | --- | --- |
| Measure | No | Yes |  | Unadjusted | Adjusted^a^ | Cohen’s d^a^ |
| Bloods, mean (sd) | (N=22) | (N=34) |  | Mean diff (95%CI) | Mean diff (95%CI) |  |
| Fasting blood insulin (pmol/L) | 94.8 (68.7) | 81.6 (47.0) |  | -13.26 (-44.23, 17.71) | -17.67 (-51.14, 15.81) | 0.31 |
| Fasting blood glucose (mmol/L) | 5.1 (0.5) | 5.2 (0.6) |  | 0.04 (-0.25, 0.33) | 0.04 (-0.26, 0.35) | 0.08 |
| Haemoglobin A1c (mmol/mol) | 33.5 (4.1) | 33.0 (4.8) |  | -0.50 (-2.98, 1.98) | -0.43 (-2.93, 2.06) | 0.10 |
| HOMA-IR | 3.0 (2.3) | 2.6 (1.6) |  | -0.41 (-1.46, 0.64) | -0.54 (-1.68, 0.61) | 0.28 |
| Blood pressure, mean (sd) | (N=22) | (N=35) |  | Mean diff (95%CI) | Mean diff (95%CI) |  |
| Systolic BP (mm Hg) | 116.0 (12.6) | 112.9 (14.5) |  | -3.10 (-10.62, 4.42) | -0.17 (-6.97, 6.62) | 0.01 |
| Diastolic BP (mm Hg) | 77.2 (10.1) | 75.6 (10.9) |  | -1.60 (-7.36, 4.16) | 0.29 (-5.47, 6.04) | 0.03 |
| Metabolic syndrome^b^, % | (N=22) | (N=34) |  | RR (95%CI) | ARR (95%CI) |  |
| Elevated waist circumference | 54.6 | 50.0 |  | 0.92 (0.55, 1.52) | 0.93 (0.58, 1.48) |  |
| Elevated triglycerides | 31.8 | 23.5 |  | 0.74 (0.31, 1.75) | 0.70 (0.29, 1.66) |  |
| Reduced HDL-C | 50.0 | 52.9 |  | 1.06 (0.63, 1.79) | 1.01 (0.58, 1.75) |  |
| Elevated BP | 13.6 | 23.5 |  | 1.73 (0.51, 5.81) | 2.76 (0.82, 9.30) |  |
| Elevated fasting glucose | 18.2 | 17.7 |  | 0.97 (0.31, 3.05) | 1.50 (0.49, 4.57) |  |
| Metabolic syndrome | 36.4 | 23.5 |  | 0.65 (0.28, 1.47) | 0.77 (0.39, 1.51) |  |

HOMA-IR: homeostatic model assessment for insulin resistance, BP: blood pressure, HDL–C: high density lipoprotein cholesterol  ^a^ Adjusted for sex, ethnicity, birthweight z-score, duration of breast feeding, parental education and potential selection bias
^b^ Symptoms of metabolic syndrome and elevated waist circumference defined according to International Diabetes Federation criteria (Alberti KG et al. Diabet Med 2006;23:469-80)

eTable 11. Visual and cardiovascular outcomes by receipt of antenatal corticosteroids (ACS) amongst extremely preterm (<28 weeks) infants

|  | Receipt of ACS | |  | Mean difference/RR (95% CI) | |  |
| --- | --- | --- | --- | --- | --- | --- |
| Measure | No | Yes |  | Unadjusted | Adjusted^a^ | Cohen’s d^a^ |
| Visual outcomes, % | (N=22) | (N=35) |  | RR (95%CI) | ARR (95%CI) |  |
| Visual acuity (logMAR) >0.3 better eye | 9.1 | 17.1 |  | 1.89 (0.42, 8.53) | 0.92 (0.28, 3.04) |  |
| Myopia >2.0 D better eye | 23.8 | 9.1 |  | 0.38 (0.10, 1.43) | 0.33 (0.09, 1.11) |  |
| Hypermetropia >2.0 D better eye | 0.0 | 0.0 |  | - | - |  |
| Astigmatism >2.0 D better eye | 4.8 | 3.0 |  | 0.63 (0.04, 9.63) | 0.70 (0.20, 2.45) |  |
| Moderate visual impairment (any of the above) | 36.4 | 25.7 |  | 0.71 (0.32, 1.56) | 0.60 (0.30, 1.20) |  |
| Heart structure and function, mean(sd) | (N=22) | (N=35) |  | Mean diff (95%CI) | Mean diff (95%CI) |  |
| LV mass - indexed BSA (g/m^2^) | 84.4 (14.4) | 83.1 (17.8) |  | -1.29 (-10.50, 7.91) | -0.29 (-10.45, 9.86) | 0.02 |
| LVEDV - indexed BSA (mL/m^2^) | 59.4 (9.8) | 56.4 (9.7) |  | -3.05 (-8.43, 2.33) | -3.68 (-9.91, 2.54) | 0.38 |
| LVESV - indexed BSA (mL/ m^2^) | 21.1 (4.3) | 19.9 (3.9) |  | -1.17 (-3.43, 1.09) | -1.26 (-3.57, 1.05) | 0.31 |
| LV elastance (mm Hg/mL) | 3.19 (0.83) | 3.56 (1.00) |  | 0.37 (-0.16, 0.90) | 0.49 (-0.10, 1.07)) | 0.52 |
| Arterial elastance (mm Hg/mL) | 1.77 (0.42) | 1.93 (0.49) |  | 0.15 (-0.11, 0.42) | 0.22 (-0.09, 0.53) | 0.47 |
| Reactive hyperaemic index (RHI)^b^ | 2.09 (0.87) | 2.00 (0.55) |  | -0.09 (-0.49, 0.32) | -0.01 (-0.41, 0.39) | 0.01 |
| Cardiac output - indexed BSA (L/min) | 2.72 (0.60) | 2.66 (0.73) |  | -0.06 (-0.44, 0.32) | -0.07 (-0.43, 0.30)) | 0.10 |

logMAR: log of minimum angle of resolution, D: dioptres, LV: left ventricular, BSA: body surface area, LVEDV: LV end-diastolic volume, LVESV: LV end-systolic volume.  ^a^ Adjusted for sex, ethnicity, birthweight z-score, duration of breast feeding, parental education and potential selection bias.
^b^ Sample sizes for RHI – no ACS (N=19), ACS (N=31)

eTable 12. Adult mental health, substance use and antisocial behaviour outcomes by antenatal corticosteroids (ACS) amongst extremely preterm (<28 weeks) infants

| Measure | Receipt of ACS | | Unadjusted RR (95% CI) | Adjusted RR (95%CI)^a^ |
| --- | --- | --- | --- | --- |
|  | No  (N=25) | Yes  (N=40) |  |  |
| Mental health (past 12 months), % |  |  |  |  |
| Major depression | 12.0 | 22.5 | 1.88 (0.56, 6.27) | 2.98 (0.90, 9.88) |
| Suicidal ideation | 8.0 | 10.0 | 1.25 (0.25, 6.33) | 1.53 (0.48, 4.89) |
| Any anxiety disorder | 20.0 | 27.5 | 1.38 (0.54, 3.49) | 1.71 (0.63, 4.63) |
| Any of the above | 24.0 | 40.0 | 1.67 (0.75, 3.69) | 2.05 (0.84, 4.99) |
| Substance use/antisocial behaviour, % |  |  |  |  |
| Daily smoker | 28.0 | 27.5 | 0.98 (0.44, 2.20) | 0.82 (0.41, 1.63) |
| Regular (weekly) binge drinking | 16.0 | 12.5 | 0.78 (0.23, 2.64) | 0.63 (0.21, 1.89) |
| Daily cannabis use | 16.0 | 17.5 | 1.09 (0.36, 3.36) | 0.83 (0.25, 2.77) |
| History of adult offending (>18 yrs) | 16.0 | 22.5 | 1.41 (0.48, 4.09) | 1.19 (0.39. 3.63) |

^a^ Adjusted for sex, ethnicity, birthweight z-score, parental education and potential selection bias

eTable 13. Adult IQ and attention deficit hyperactivity disorder (ADHD) by receipt of antenatal corticosteroids (ACS) amongst extremely preterm (<28 weeks) infants

| Measure | Receipt of ACS | |  | Mean difference (95% CI) | |  |
| --- | --- | --- | --- | --- | --- | --- |
|  | No | Yes |  | Unadjusted | Adjusted^a^ | Cohen’s d^a^ |
| WASI II IQ scores, mean (sd) | (N=22) | (N=35) |  |  |  |  |
| Verbal IQ | 96.7 (13.5) | 102.9 (15.3) |  | 6.23 (-1.75, 14.21) | 5.44 (-1.57, 12.45) | 0.37 |
| Perceptual IQ | 94.2 (13.0) | 97.7 (9.1) |  | 3.52 (-2.52, 9.56) | 3.41 (-3.08, 9.91) | 0.32 |
| Total IQ | 95.6 (11.6) | 100.3 (11.7) |  | 4.65 (-1.87, 11.17) | 4.04 (-2.16, 10.23) | 0.34 |
| Adult ADHD, mean (sd) | (N=23) | (N=34) |  |  |  |  |
| ADHD symptom score | 9.1 (8.0) | 9.2 (7.2) |  | 0.08 (-4.02, 4.17) | -0.53 (-5.34, 4.27) | 0.07 |

WASI II: Wechsler Abbreviated Scale of Intelligence – Version II, ADHD: Attention Deficit Hyperactivity Disorder
^a^ Adjusted for sex, ethnicity, birthweight z-score, gestation, duration of breast feeding, parental education and potential selection bias

eTable 14. Adjusted^a^ effect size estimates for antenatal corticosteroids (ACS) from analyses excluding gestation, birthweight z-score as covariates. Effect sizes reported as mean difference (MD) (95% CI) and Cohen’s d or adjusted rate ratio (ARR) (95%CI).

| Measure |  | Total Sample | | |  | Females | |  | Males | |  | Extremely Preterm | |
| --- | --- | --- | --- | --- | --- | --- | --- | --- | --- | --- | --- | --- | --- |
| Adult growth outcomes |  | MD (95% CI) | | d |  | MD (95% CI) | d |  | MD (95% CI) | d |  | MD (95% CI) | d |
| Height, cm | | 0.60 (-1.08, 2.28) | | 0.07 |  | -0.68 (-3.04, 1.68) | 0.11 |  | 1.53 (1.01, 4.06) | 0.26 |  | -2.20 (-5.74, 1.34) | 0.28 |
| Weight, kg | | 1.37 (-3.61, 6.34) | | 0.07 |  | -2.41 (-9.52, 1.72) | 0.13 |  | 4.62 (-2.68, 11.92) | 0.26 |  | -4.63 (-16.37, 7.12) | 0.22 |
| BMI, kg/m^2^ | | 0.38 (-1.35, 1.95) | | 0.06 |  | -0.63 (-3.09, 1.84) | 0.09 |  | 1.21 (-1.07, 3.48) | 0.22 |  | -0.97 (5.03, 3.10) | 0.14 |
| Waist circumference, cm | | 0.66 (-3.17, 4.48) | | 0.04 |  | -0.92 (-6.15, 4.32) | 0.06 |  | 1.90 (-4.08, 7.89) | 0.13 |  | -2.34 (12.28, 7.61) | 0.13 |
| Hip circumference, cm | | 1.63 (-1.64, 4.90) | | 0.13 |  | -0.60 (-5.62, 4.42) | 0.04 |  | 3.74 (-0.53, 8.01) | 0.37 |  | -2.18 (-10.40, 6.03) | 0.16 |
| Waist/hip ratio | | -0.01 (-0.03, 0.01) | | 0.11 |  | -0.01 (-0.04, 0.02) | 0.12 |  | -0.1 (-0.05, 0.02) | 0.13 |  | 0.00 (-0.06, 0.06) | 0.00 |
| Body fat percentage, % | | -0.37 (-2.88, 2.14) | | 0.03 |  | -1.26 (-4.97,2.45) | 0.13 |  | 0.09 (-3.52, 3.70) | 0.01 |  | -1.62 (-7.30, 4.07) | 0.15 |
| Fat mass, kg | | 0.39 (-3.32, 4.09) | | 0.03 |  | -1.67 (-7.12, 3.78) | 0.12 |  | 1.78 (-3.53, 7.09) | 0.14 |  | -1.80 (-10.06, 6.47) | 0.12 |
| Fat free mass, kg | | 1.22 (-0.59, 3.04) | | 0.12 |  | -1.10 (-3.27, 1.07) | 0.20 |  | 3.48 (0.41, 6.55) | 0.47 |  | -0.69 (-5.06, 3.68) | 0.07 |
| Total body water, kg | | 0.90 (-0.43, 2.22) | | 0.12 |  | -0.80 (-2.38, 0.79) | 0.20 |  | 2.54 (0.30, 4.79) | 0.47 |  | -0.46 (3.69, 2.77) | 0.06 |
| Bloods | | MD (95% CI) | | d |  | MD (95% CI) | d |  | MD (95% CI) | d |  | MD (95% CI) | d |
| Fasting blood insulin  (pmol/L) | | -1.53  (-17.18, 14.13) | | 0.03 |  | -10.49  (-26.01, 5.02) | 0.20 |  | 10.67  (-19.86, 41.22) | 0.15 |  | -17.64  (-50.45, 15.17) | 0.31 |
| Fasting blood glucose  (mmol/L) | | 0.06 (-0.08, 0.20) | | 0.12 |  | 0.07 (-0.13, 0.27) | 0.14 |  | 0.04 (-0.17, 0.26) | 0.08 |  | 0.06 (-0.24, 0.37) | 0.11 |
| Haemoglobin A1c   (mmol/mol) | | 1.09 (-0.08, 2.26) | | 0.25 |  | 1.51 (-0.19, 3.22) | 0.32 |  | 0.78 (-0.90, 2.46) | 0.19 |  | -0.33 (-2.82, 2.14) | 0.07 |
| HOMA-IR | | -0.01 (-0.53,0.51) | | 0.01 |  | -0.28 (-0.80, 0.24) | 0.19 |  | 0.36 (-0.65, 1.37) | 0.16 |  | -0.53 (1.65, 0.59) | 0.28 |
| Blood pressure | | MD (95% CI) | | d |  | MD (95% CI) | d |  | MD (95% CI) | d |  | MD (95% CI) | d |
| Systolic BP (mm Hg) | | -0.10 (-3.29, 3.09) | | 0.01 |  | -1.37 (-5.58, 2.85) | 0.12 |  | -0.12 (-5.23, 4.99) | 0.01 |  | -1.16 (-8.28, 5.96) | 0.08 |
| Diastolic BP (mm Hg) | | 0.50 (-1.89, 2.89) | | 0.05 |  | 0.57 (-2.54, 3.67) | 0.07 |  | -0.54 (-4.43, 3.36) | 0.06 |  | 0.34 (-6.21, 5.53) | 0.03 |
| Metabolic syndrome | | ARR (95% CI) | |  |  | ARR (95% CI) |  |  | ARR (95% CI) |  |  | ARR (95% CI) |  |
| Elevated waist   circumference | | 1.11 (0.79, 1.56) | |  |  | 1.00 (0.69, 1.47) |  |  | 1.34 (0.66, 2.71) |  |  | 0.89 (0.55, 1.43) |  |
| Elevated triglycerides | | 0.81 (0.47, 1.39) | |  |  | 0.45 (0.18, 1.12) |  |  | 1.16 (0.58, 2.32) |  |  | 0.71 (0.30, 1.66) |  |
| Reduced HDL-C | | 1.17 (0.84, 1.63) | |  |  | 1.02 (0.70, 1.49) |  |  | 1.63 (0.83, 3.20) |  |  | 1.02 (0.59, 1.78) |  |
| Elevated BP | | 0.97 (0.54, 1.72) | |  |  | 1.42 (0.36, 5.60) |  |  | 0.78 (0.40, 1.54) |  |  | 2.69 (0.86, 8.44) |  |
| Elevated fasting   glucose | | 1.86 (0.93, 3.74) | |  |  | 1.56 (0.63, 5.50) |  |  | 1.51 (0.63, 3.67) |  |  | 1.51 (0.49, 4.64) |  |
| Metabolic syndrome | | 0.88 (0.9, 1.60) | |  |  | 0.51 (0.18, 1.45) |  |  | 1.19 (0.55, 2.58) |  |  | 0.75 (0.37, 1.51) |  |
| Visual outcomes | | ARR (95% CI) | |  |  | ARR (95% CI) |  |  | ARR (95% CI) |  |  | ARR (95% CI) |  |
| Visual acuity (logMAR)  >0.3 better eye | | 1.88 (0.64, 5.51) | |  |  | 0.60 (0.19, 1.89) |  |  | 6.97 (0.96, 50.46) |  |  | 0.99 (0.29, 3.38) |  |
| Myopia >2.0 D better  eye | | 0.61 (0.32, 1.16) | |  |  | 0.73 (0.34, 1.60) |  |  | 0.40 (0.09, 1.80) |  |  | 0.36 (0.11, 1.24) |  |
| Astigmatism >2.0 D  better eye | | 0.76 (0.27, 2.12) | |  |  | 0.22 (0.05, 0.95) |  |  | 1.69 (0.45, 6.34) |  |  | -^b^ |  |
| Moderate visual impairment | | 1.01 (0.61, 1.67) | |  |  | 0.70 (0.36, 1.35) |  |  | 1.39 (0.64, 3.01) |  |  | 0.61 (0.30, 1.27) |  |
| Heart structure and function | | | MD (95% CI) | d |  | MD (95% CI) | d |  | MD (95% CI) | d |  | MD (95% CI) | d |
| LV mass - indexed BSA   (g/m^2^) | | -0.33 (-5.44, 4.78) | | 0.02 |  | -5.29 (-17.06, 1.47) | 0.30 |  | 5.19 (-2.87, 13.24) | 0.28 |  | -1.81 (-12.22, 8.60) | 0.11 |
| LVEDV - indexed BSA  (mL/m^2^) | | -0.63 (-3.61, 2.34) | | 0.06 |  | -0.81 (-4.40, 2.78) | 0.09 |  | -1.15 (-6.34, 4.04) | 0.10 |  | -3.84 (-9.95, 2.27) | 0.39 |
| LVESV - indexed BSA   (mL/ m^2^) | | -0.39 (-1.68, 0.90) | | 0.08 |  | -0.16 (-1.67, 1.35) | 0.04 |  | -0.99 (-3.29, 1.30) | 0.19 |  | -1.49 (-3.97, 0.98) | 0.37 |
| LV elastance (mm   Hg/mL) | | 0.09 (-0.15, 0.32) | | 0.10 |  | 0.29 (-0.06, 0.64) | 0.33 |  | -0.07 (-0.40, 0.27) | 0.09 |  | 0.55 (-0.05, 1.14) | 0.58 |
| Arterial elastance (mm   Hg/mL) | | 0.03 (-0.09, 0.14) | | 0.07 |  | 0.14 (-0.02, 0.30) | 0.34 |  | -0.07 (-0.24, 0.09) | 0.19 |  | 0.23 (-0.08, 0.53) | 0.49 |
| RHI^b^ | | -0.03 (-0.20, 0.14) | | 0.05 |  | 0.09 (-0.14, 0.32) | 0.15 |  | -0.16 (-0.43, 0.10) | 0.28 |  | -0.07 (-0.47, 0.33) | 0.10 |
| Cardiac output-   indexed BSA (L/min) | | 0.01 9-0.16, 0.19) | | 0.02 |  | 0.04 (-0.19, 0.27) | 0.06 |  | -0.05 (-0.34, 0.24) | 0.08 |  | -0.01 (-0.37. 0.35) | 0.01 |
| Mental health (past 12 mths) | | | ARR (95% CI) |  |  | ARR (95% CI) |  |  | ARR (95% CI) |  |  | ARR (95% CI) |  |
| Major depression | | 2.03 (0.98, 4.21) | |  |  | 2.46 (0.76, 8.00) |  |  | 1.53 (0.59, 3.98) |  |  | 2.76 (0.80, 9.53) |  |
| Suicidal ideation | | 1.37 (0.45, 4.14) | |  |  | 1.31 (0.76, 2.26) |  |  | 0.51 (0.11, 2.31) |  |  | 1.31 (0.20, 8.67) |  |
| Anxiety disorder | | 1.06 (0.69, 1.64) | |  |  | 1.25 (0.74, 2.10) |  |  | 0.87 (0.37, 2.05) |  |  | 1.64 (0.68, 4.07) |  |
| Any of the above | | 1.20 (0.82, 1.74) | |  |  | 1.28 (0.79, 2.07) |  |  | 1.06 (0.57, 1.98) |  |  | 1.88 (0.79, 4.48) |  |
| Substance use/ antisocial behavior | | | ARR (95% CI) |  |  | ARR (95% CI) |  |  | ARR (95% CI) |  |  | ARR (95% CI) |  |
| Daily smoker | | 1.08 (0.71, 1.63) | |  |  | 0.93 (0.53, 1.65) |  |  | 1.18 (0.65, 2.13) |  |  | 0.82 (0.62, 1.60) |  |
| Regular (weekly) binge  drinking | | 0.85 (0.47, 1.56) | |  |  | 1.05 (0.43, 2.55) |  |  | 0.69 (0.2, 1.68) |  |  | 0.63 (0.20, 1.97) |  |
| Daily cannabis use | | 1.06 (0.39, 2.86) | |  |  | 2.13 (0.36, 12.62) |  |  | 0.83 (0.27, 2.52) |  |  | 0.85 (0.27, 2.70) |  |
| History of adult  offending (>18 yrs) | | 0.69 (0.421, 1.14) | |  |  | 0.73 (0.33, 1.62) |  |  | 0.75 (0.39, 1.47) |  |  | 1.18 (0.36, 3.85) |  |
| WASI II IQ and Adult ADHD | | MD (95% CI) | | d |  | MD (95% CI) | d |  | MD (95% CI) | d |  | MD (95% CI) | d |
| Verbal IQ | | 0.49 (-3.01, 3.99) | | 0.04 |  | 1.71 (-2.47, 5.88) | 0.12 |  | -1.94 (-8.13, 4.26) | 0.14 |  | 5.11 (-1.85, 12.06) | 0.34 |
| Perceptual IQ | | 2.40 (-2.11, 6.90) | | 0.16 |  | 1.06 (-3.81, 5.93) | 0.08 |  | 3.20 (-5.22, 11.63) | 0.19 |  | 2.85 (-3.80, 9.50) | 0.26 |
| Total IQ | | 1.52 (-2.46, 5.51) | | 0.11 |  | 1.63 (-2.67, 5.93) | 0.12 |  | 0.53 (-6.90, 7.98) | 0.03 |  | 3.55 (-2.75, 9.85) | 0.30 |
| ADHD symptom score | | 0.29 (-1.73, 3.00) | | 0.04 |  | 0.60 (-2.28, 3.49) | 0.01 |  | -0.08 (-2.88, 2.72) | 0.01 |  | -0.83 (-5.72, 4.06) | 0.11 |

BMI: body mass index, HOMA-IR: homeostatic model assessment for insulin resistance, BP: blood pressure, HDL–C: high density lipoprotein cholesterol, logMAR: log of minimum angle of resolution, D: dioptres, LV: left ventricular, BSA: body surface area, LVEDV: LV end-diastolic volume, LVESV: LV end-systolic volume, RHI: reactive hyperaemic index, WASI II: Wechsler Abbreviated Scale of Intelligence – Version II, ADHD: Attention Deficit Hyperactivity Disorder

^a^ Adjusted for sex, ethnicity, parental education, duration of breastfeeding, and potential selection bias
^b^ Model not estimable

eFigure 1. New Zealand 1986 VLBW Adult Follow-up Study: Cohort flow chart

Recorded as live born with birth weight <1500g and admitted to a
neonatal unit in New Zealand in 1986
*n*=413

Included in prospective cohort audit of retinopathy of prematurity
*n*=413

Died before discharge from NICU *n*=75
Died between discharge and 7 years *n*=12

Alive at 26 years and eligible for follow-up *n*=323

NOT ASSESSED at 26-30 years *n*=73
Not traced *n=35*
Declined *n=38*

ASSESSED at 26-30 years *n*=250

Full assessment over 2 days^a^
(including 29 living overseas) *n*=229

Assessment by questionnaire only^a^ *n*=21

Died between 8 and 22 years *n*=3

Alive at 7 years and eligible for follow-up *n*=326

NOT ASSESSED at age 7-8 years *n*=28
Overseas/ not approached *n*=17
Lost to follow-up *n=*4
Declined *n*=7

ASSESSED at 7-8 years
at a home visit *n*=298

Alive at 22 years and eligible for follow-up *n*=323

NOT ASSESSED at 22-23 years *n*=93

Not traced *n*=72

Declined *n*=21

ASSESSED at 22-23 years
at face-to-face interview *n*=230

Died between 22 and 26 years *n*=0

^a^Between February 2013 and November 2016

NICU: Neonatal intensive care unit

[Modified, with permission from Darlow BA, et al. Metabolic syndrome in very low birth weight young adults and controls: the New Zealand 1986 VLBW Study. *J Pediatr* 2019;206:128-33 (133.e1)]
